# Bayesian network model of ethno-racial disparities in cardiometabolic-based chronic disease using NHANES 1999–2018

**DOI:** 10.3389/fpubh.2024.1409731

**Published:** 2024-10-15

**Authors:** Masih A. Babagoli, Michael J. Beller, Juan P. Gonzalez-Rivas, Ramfis Nieto-Martinez, Faris Gulamali, Jeffrey I. Mechanick

**Affiliations:** ^1^Icahn School of Medicine at Mount Sinai, New York, NY, United States; ^2^Beller Tech LLC, New York, NY, United States; ^3^Department of Global Health and Population, Harvard TH Chan School of Public Health, Boston, MA, United States; ^4^Foundation for Clinic, Public Health, and Epidemiology Research of Venezuela (FISPEVEN INC), Caracas, Venezuela; ^5^International Clinical Research Center (ICRC), St. Anne's University Hospital, Brno, Czechia; ^6^Precision Care Clinic Corp, Saint Cloud, Saint Cloud, FL, United States; ^7^The Marie-Josée and Henry R. Kravis Center for Cardiovascular Health at Mount Sinai Fuster Heart Hospital, New York, NY, United States; ^8^Division of Endocrinology, Diabetes and Bone Disease, Icahn School of Medicine at Mount Sinai, New York, NY, United States

**Keywords:** cardiometabolic disease, health inequities, racial inequities, social determinants of health, obesity

## Abstract

**Background:**

Ethno-racial disparities in cardiometabolic diseases are driven by socioeconomic, behavioral, and environmental factors. Bayesian networks offer an approach to analyze the complex interaction of the multi-tiered modifiable factors and non-modifiable demographics that influence the incidence and progression of cardiometabolic disease.

**Methods:**

In this study, we learn the structure and parameters of a Bayesian network based on 20 years of data from the US National Health and Nutrition Examination Survey to explore the pathways mediating associations between ethno-racial group and cardiometabolic outcomes. The impact of different factors on cardiometabolic outcomes by ethno-racial group is analyzed using conditional probability queries.

**Results:**

Multiple pathways mediate the indirect association from ethno-racial group to cardiometabolic outcomes: (1) ethno-racial group to education and to behavioral factors (diet); (2) education to behavioral factors (smoking, physical activity, and—via income—to alcohol); (3) and behavioral factors to adiposity-based chronic disease (ABCD) and then other cardiometabolic drivers. Improved diet and physical activity are associated with a larger decrease in probability of ABCD stage 4 among non-Hispanic White (NHW) individuals compared to non-Hispanic Black (NHB) and Hispanic (HI) individuals.

**Conclusion:**

Education, income, and behavioral factors mediate ethno-racial disparities in cardiometabolic outcomes, but traditional behavioral factors (diet and physical activity) are less influential among NHB or HI individuals compared to NHW individuals. This suggests the greater contribution of unmeasured individual- and/or neighborhood-level structural determinants of health that impact cardiometabolic drivers among NHB and HI individuals. Further study is needed to discover the nature of these unmeasured determinants to guide cardiometabolic care in diverse populations.

## Introduction

The prevalence of cardiometabolic-based chronic diseases—including obesity, type 2 diabetes (T2D), and cardiovascular disease (CVD) phenotypes—is rapidly increasing both in the US and globally (1). The incidence and progression of these conditions are determined by a complex interaction of nonmodifiable genetic and demographic factors with modifiable socioeconomic, behavioral, and environmental factors (2, 3). In the US, there are significant disparities in the prevalence of obesity (4), T2D (5), hypertension (6), hyperlipidemia (7), and CVD (8) across ethno-racial groups. Given that race is a socially constructed designation, the source of these ethno-racial disparities is through socioeconomic, behavioral, and environmental variables influenced by policies and structural racism (3). Hence, there is a need to better understand the intermediate pathways by which various ethno-racial group descriptors influence relevant cardiometabolic health outcomes in order to better address health disparities (2).

Multiple pathways connecting ethno-racial group and cardiometabolic health disparities have been reported in terms of associations among intermediate variables (3). However, there are limited studies evaluating the interactions of multiple tiers of variables using a network approach. For instance, van den Houdt et al. (9) created a mixed graphical model based on biomedical, behavioral, psychosocial, and socioeconomic variables based on a Dutch cohort study of patients undergoing percutaneous coronary intervention and showed that socioeconomic status had a central position in their network. Ordovas et al. (10) created a Bayesian network based on expert opinion and a Spanish insurance dataset to derive a predictive CVD risk tool. Fuster-Parra et al. (11, 12) created a Bayesian network based on a Spanish worker dataset and detailed the impact of demographic and behavioral factors on specific CVD and diabetes outcomes. However, data from quantitative network analyses to understand the impact of ethno-racial groupings on cardiometabolic health outcomes as well as the respective intermediate pathways mediating ethno-racial disparities are lacking.

Bayesian networks are a class of probabilistic graphical models that can represent a network of variables (nodes) and the conditional dependencies between those variables (edges) as a directed acyclic graph (DAG) (13–15). Bayesian networks have been used in a variety of analyses to study cardiometabolic disease (10, 11, 16) and related questions (17, 18). The structure and parameters of Bayesian networks can be learned from large datasets and/or incorporate *a priori* knowledge of relationships among variables. Additionally, Bayesian networks are able to capture non-linear associations between nodes and also can be utilized to conduct queries to estimate the probability of a target variable given values of other variables.

In this study, we construct a Bayesian network model based on 20 years of data from the US National Health and Nutrition Examination Survey (NHANES) to: (1) analyze potential pathways that mediate ethno-racial disparities in cardiometabolic health outcomes; (2) understand how the underlying network of variables influencing cardiometabolic health outcomes differs by ethno-racial group; and (3) analyze the differential impact of behavioral factors on cardiometabolic health outcomes by ethno-racial group. We interpret cardiometabolic health using the four-stage (risk, predisease, disease, and complications) cardiometabolic-based chronic disease (CMBCD) model designed to expose early opportunities for preventive care (19). The CMBCD model incorporates three primary drivers (genetics, environment, and behavior) and four secondary drivers [adiposity-based chronic disease (ABCD), dysglycemia-based chronic disease (DBCD), hypertension-based chronic disease (HBCD), and lipid-based chronic disease (LBCD)] of specific CVDs (atherosclerosis, heart failure, and atrial fibrillation) (19–22). Overall, the use of Bayesian networks to analyze a large dataset of multi-tiered variables influencing a staged measure of cardiometabolic health provides a unique opportunity to understand cardiometabolic health disparities.

## Methods

### Data source

This study is a secondary analysis of data collected as part of NHANES over 10 survey cycles between 1999 and 2018. Since 1999, NHANES has been conducted on an ongoing basis, and the data has been released in two-year cycles. The specifics of the survey design have been described elsewhere (23). Briefly, NHANES uses a stratified, clustered four-stage sampling approach to collect cross-sectional data representative of the non-institutionalized civilian population living in the 50 US states and the District of Columbia. Collected information included demographic, health, and nutrition variables, as well as select laboratory measurements.

### Model variables

Model variables were chosen based on three criteria: (1) relation to socio-demographics, social determinants of health (SDOH), behavioral factors, and cardiometabolic health outcomes; (2) association with cardiometabolic health; and (3) inclusion of relevant measures in NHANES across the 10 survey cycles between 1999 and 2018. There were 18 variables chosen for the network: age, gender, ethno-racial group, education, income, employment status, household food security, health insurance, routine healthcare site, diet, physical activity, alcohol use, smoking status, ABCD, DBCD, HBCD, LBCD, and CMBCD. These 18 variables were defined based on appropriate NHANES dataset variables, and any differences in how the questions were asked or reported across the 10 survey cycles were harmonized. In all cases, the variables were made categorical, which was accomplished by applying appropriate categorical thresholds to continuous variables, where necessary.

Cardiometabolic health was formulated using the CMBCD model, which incorporates four secondary drivers of cardiometabolic disease – ABCD, DBCD, HBCD, and LBCD (19–22). For ABCD, DBCD, HBCD, and LBCD, each individual was classified for each driver as stage 0 (no disease without the presence of risk factors), stage 1 (no disease with the presence of behavioral or metabolic risk factors), stage 2 (predisease), stage 3 (disease), and stage 4 (pre-disease with complications or disease with complications) (19–22). In defining CMBCD, the following types of CVD were used: congestive heart failure, coronary artery disease, heart attack, and stroke. Due to lack of any NHANES data that could differentiate predisease, disease, and complications for the aforementioned CVDs, CMBCD was categorized as stage 0 (no history of CVD and all cardiometabolic drivers in stage 0), stage 1 (no history of CVD and any cardiometabolic driver in stage 1 or higher), and stage 2–4 (history of CVD with any cardiometabolic driver in stage 1 or higher) (19) (see Supplementary Table 1 for full definitions of model variables).

The model variables were organized into the following tiers in descending order: non-modifiable socio-demographics (age, gender, and ethno-racial group), modifiable socio-demographics (education, income, employment status), SDOH (household food security, health insurance, and routine healthcare site), behavioral factors (diet, physical activity, alcohol use, and smoking status), secondary cardiometabolic drivers (ABCD, DBCD, HBCD, and LBCD), and CMBCD. Exclusion criteria for this analysis were missing values for any of the model variables, age less than 20 years old, being pregnant, and presence of type 1 diabetes.

### Bayesian network learning

Bayesian networks are a class of probabilistic graphical models that consist of variables (nodes) and the conditional dependencies between those variables (directed edges) as a DAG (13–15). For a DAG *G* with parameters *Θ*, the joint probability distribution *P* over a vector of random variables *X = (X_1_, …, X_n_)* can be formulated as


$$P\left(X|G,\Theta \right)=\overset{n}{\underset{i=1}{\prod }}P\left(X_{i}|pa\left(X_{i}\right)\right)$$

Thus, the joint probability distribution is the product of the individual conditional probabilities for each variable X_i_ given the probability of its parent nodes pa (X_i_) (14).

Bayesian network learning consists of two steps—structure learning of the DAG followed by parameter learning. Structure learning of the network edges in the DAG was conducted based on the NHANES dataset using the hill-climbing algorithm, which has been commonly used in similar analyses and described elsewhere (11, 12, 24–26). Briefly, hill-climbing is a score-based graph structure learning algorithm that explores the space of possible DAGs through sequential arc addition, deletion, and reversal steps with the goal of optimizing goodness of fit in order to find the optimal DAG structure (24, 25). By stipulating blacklists and whitelists in the structure learning step, the network was subject to the following constraints to integrate *a priori* knowledge and improve interpretability of the network: (1) no edge from a variable in a given tier to another variable in a higher tier (for example, no edge allowed from diet to income); (2) no edge from any variable to the non-modifiable socio-demographic variables (age, gender, and ethno-racial group); (3) no edge from ethno-racial group directly to ABCD, DBCD, HBCD, LBCD, or CMBCD; (4) presence of edges among ABCD, DBCD, HBCD, and LBCD based on the pre-defined hierarchy representing known relationships between cardiometabolic drivers; and (5) edges from ABCD, DBCD, HBCD, and LBCD to CMBCD based on the definition of CMBCD (see Supplementary Tables 2a,b for list of specific network constraints) (19). The learned network edges were validated by bootstrap resampling the data (*n* = 200) and constructing an averaged consensus network based on edges with a probability greater than a specified threshold. This bootstrap strength confidence/significance threshold was determined by the *bnlearn* package using a statistically motivated approach that has been shown to outperform *ad hoc* thresholds in terms of sensitivity while maintaining comparable specificity and accuracy (27, 28). This approach has been described in-depth elsewhere; briefly, the edge significance threshold is chosen by minimizing the L_1_ norm between the cumulative distribution function of the observed edge significance levels and those of its asymptotic counterpart (28). Finally, learning of the network parameters was conducted using the NHANES dataset without incorporation of any *a priori* values.

The dataset was then stratified by ethno-racial group, and Bayesian networks were learned based on the same steps outlined above for the overall network. Separate Bayesian networks were learned for each of four ethno-racial groups included in NHANES [non-Hispanic White (NHW), non-Hispanic Black (NHB), Hispanic (HI), and other race or multiracial (OM)]. The authors acknowledge that race and ethnicity are different concepts and that there are ethno-racial groups beyond the four self-identified in NHANES. However, these four ethno-racial groups were used in this analysis because NHANES only reports a single variable combining race and ethnicity with the four aforementioned categories. Even though post-2011 NHANES also reports “non-Hispanic Asian” separate from “other race or multiracial,” this distinction was not used here in order to allow a consistent categorization with pre-2011 NHANES.

Given the learned network structure for each ethno-racial group, model parameters were fit to the Bayesian networks based on the NHANES dataset. Subsequently, conditional probability queries were conducted by instantiating specific model variables and estimating cardiometabolic driver probabilities using the “likelihood weighting” method, which is an approximate inference algorithm based on Monte Carlo sampling. First, conditional probability queries were conducted by instantiating each combination of values for age, gender, and ethno-racial group to estimate the probability of each stage of ABCD, DBCD, HBCD, LBCD, and CMBCD. Second, conditional probability queries were conducted by instantiating each combination of values for age, gender, and ethno-racial group along with one of the behavioral factors (diet, physical activity, smoking status, and alcohol use) to estimate the probability of ABCD stage 4, DBCD stage 4, HBCD stage 4, LBCD stage 4, and CMBCD stage 2–4; this was intended to analyze the impact of changing values of behavioral variables on the cardiometabolic drivers for each specific combination of age, gender, and ethno-racial group. For precise estimates, 10^7^ samples were generated for each query.

All analyses were conducted using R Statistical Software (v4.2.2). Bayesian network analyses were conducted using the *bnlearn* package, and the networks were visualized using the *bnviewer* package. R code used for Bayesian network learning and analyses are included in the Supplementary materials.

### Ethical considerations

The NHANES study is approved by the National Center for Health Statistics Ethics Review Board. The public-use data is available for download at www.cdc.gov/nchs/nhanes/index.htm. No individually identifying information was accessed during this analysis.

## Results

### Overall network analysis

**Figure 1:**
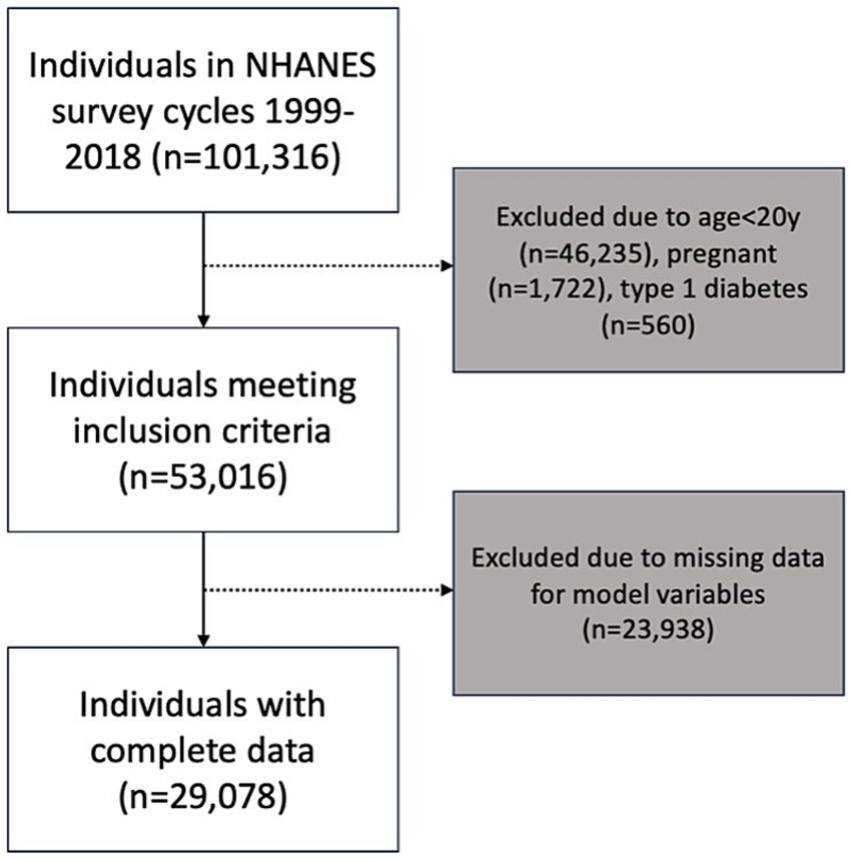
Number of participants included in analysis and reasons for exclusion.

A total of 101,316 individuals participated in the 10 NHANES survey cycles between 1999 and 2018. After applying the exclusion criteria for this analysis, the sample consisted of 53,016 individuals. The final network analysis was conducted based on a sample of 29,078 individuals who had no missing data for any of the 18 variables in the model (Figure 1). Summary statistics of composite NHANES sample used for Bayesian network learning are shown in Table 1. Note that these figures are unweighted and cannot be interpreted as nationally representative prevalence estimates.

**Table 1:** Summary statistics of composite NHANES sample (*n* = 29,078) used for Bayesian network learning.

Variable	*n*	Percent	Variable	*n*	Percent
Age			Light	8,275	28.5%
20–39	9,098	31.3%	Moderate	7,738	26.6%
40–59	9,622	33.1%	Heavy	3,570	12.3%
60+	10,358	35.6%	Smoking status		
Gender			Never	15,324	52.7%
Male	14,677	50.5%	Former	7,098	24.4%
Female	14,401	49.5%	Current	6,656	22.9%
Ethno-racial group			Physical activity		
Non-Hispanic White	14,191	48.8%	None	6,898	23.7%
Non-Hispanic Black	5,711	19.6%	Not sufficient	4,954	17.0%
Hispanic	6,856	23.6%	Sufficient	17,226	59.2%
Other or Multiracial	2,320	8.0%	Diet		
Education			Not DASH-accordant	25,404	87.4%
Less than high school	7,169	24.7%	DASH-accordant	3,674	12.6%
High school grad/GED	6,846	23.5%	ABCD		
Some college or AA degree	8,560	29.4%	Stage 0	780	2.7%
College grad or above	6,503	22.4%	Stage 1	7,485	25.7%
Income			Stage 2	1,534	5.3%
<100% FPL	5,428	18.7%	Stage 3	1,013	3.5%
100–199% FPL	7,762	26.7%	Stage 4	18,266	62.8%
200–399% FPL	7,925	27.3%	DBCD		
>400% FPL	7,963	27.4%	Stage 0	597	2.1%
Employment status			Stage 1	17,518	60.2%
Employed, homemaker, or student	18,980	65.3%	Stage 2	588	2.0%
Retired	6,389	22.0%	Stage 3	198	0.7%
Unemployed or unable to work	3,709	12.8%	Stage 4	10,177	35.0%
Household food security			HBCD		
Full	21,272	73.2%	Stage 0	326	1.1%
Marginal	2,832	9.7%	Stage 1	9,289	31.9%
Low	3,112	10.7%	Stage 2	2,694	9.3%
Very low	1862	6.4%	Stage 3	12,227	42.0%
Health insurance status			Stage 4	4,542	15.6%
No health insurance	5,729	19.7%	LBCD		
Private	16,304	56.1%	Stage 0	363	1.2%
Public - Medicare	3,892	13.4%	Stage 1	16,175	55.6%
Public - other	3,153	10.8%	Stage 2	7,821	26.9%
Routine healthcare site			Stage 3	3,662	12.6%
No routine healthcare site	4,285	14.7%	Stage 4	1,057	3.6%
Routine site - ED	793	2.7%	CMBCD		
Routine site - not ED	24,000	82.5%	Stage 0	220	0.8%
Alcohol use			Stage 1	25,704	88.4%
None	9,495	32.7%	Stage 2–4	3,154	10.8%

Note that these figures are unweighted and cannot be interpreted as nationally representative prevalence estimates. See Supplementary Table 1 for full definitions of model variables. FPL, federal poverty line; ED, emergency department, DASH, Dietary Approaches to Stop Hypertension; ABCD, adiposity-based chronic disease; DBCD, dysglycemia-based chronic disease; HBCD, hypertension-based chronic disease; LBCD, lipid-based chronic disease; CMBCD, cardiometabolic-based chronic disease.

**Figure 2:**
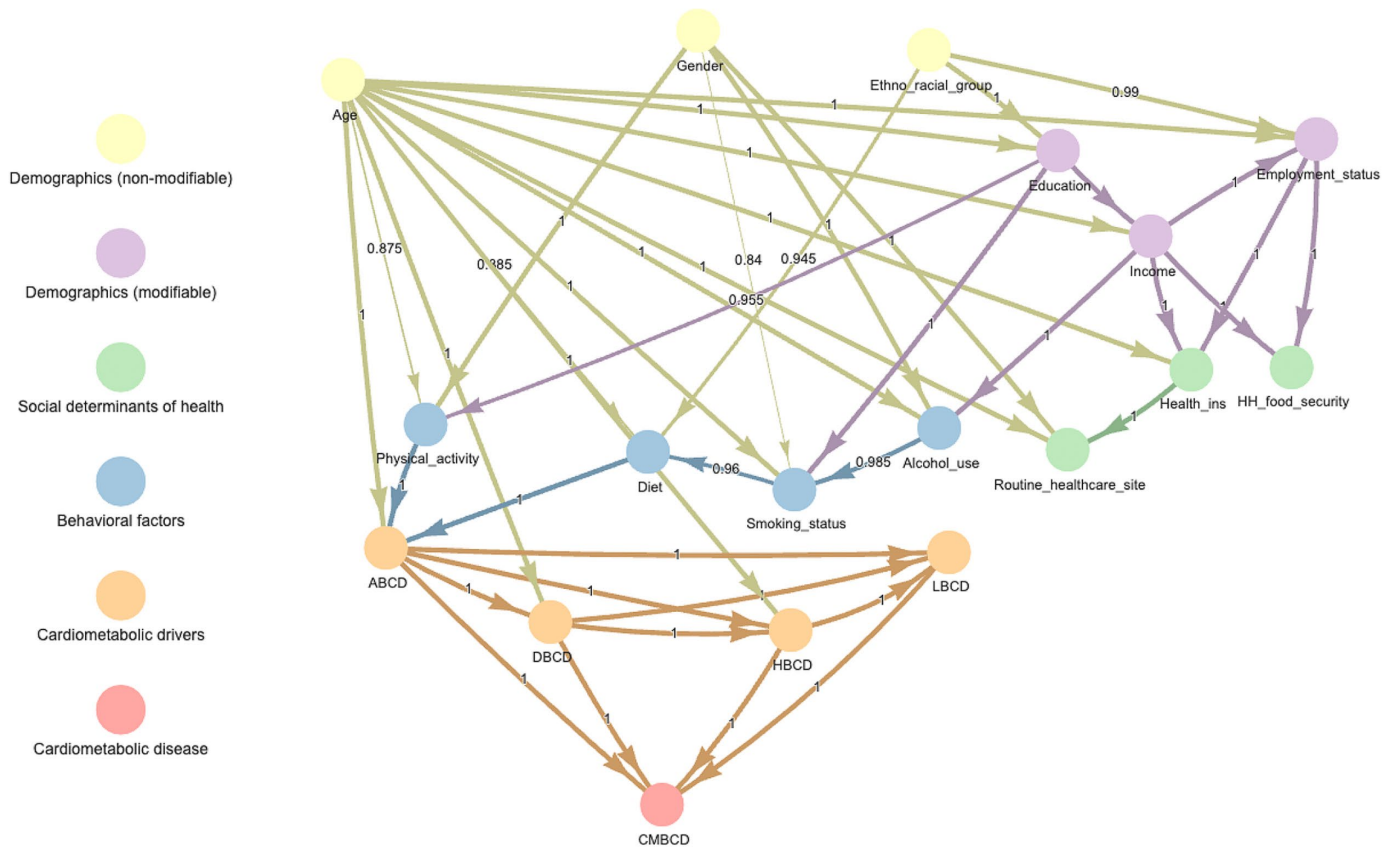
Averaged consensus Bayesian network learned from entire sample of NHANES 1999–2018. Number in middle of arrow and thickness of arrow indicate proportion of bootstrapped samples which included that relationship. ABCD, adiposity-based chronic disease; DBCD, dysglycemia-based chronic disease; HBCD, hypertension-based chronic disease; LBCD, lipid-based chronic disease; CMBCD, cardiometabolic-based chronic disease.

The Bayesian network learned from the entire sample is shown in Figure 2. In this network, there were four pathways that mediated the indirect association between ethno-racial group and the cardiometabolic outcomes: (1) ethno-racial group ➔ diet ➔ ABCD, (2) ethno-racial group ➔ education ➔ smoking status ➔ diet ➔ ABCD, (3) ethno-racial group ➔ education ➔ physical activity ➔ ABCD, and (4) ethno-racial group ➔ education ➔ income ➔ alcohol use ➔ smoking status ➔ diet ➔ ABCD.

### Network analysis by ethno-racial group

**Figure 3:**
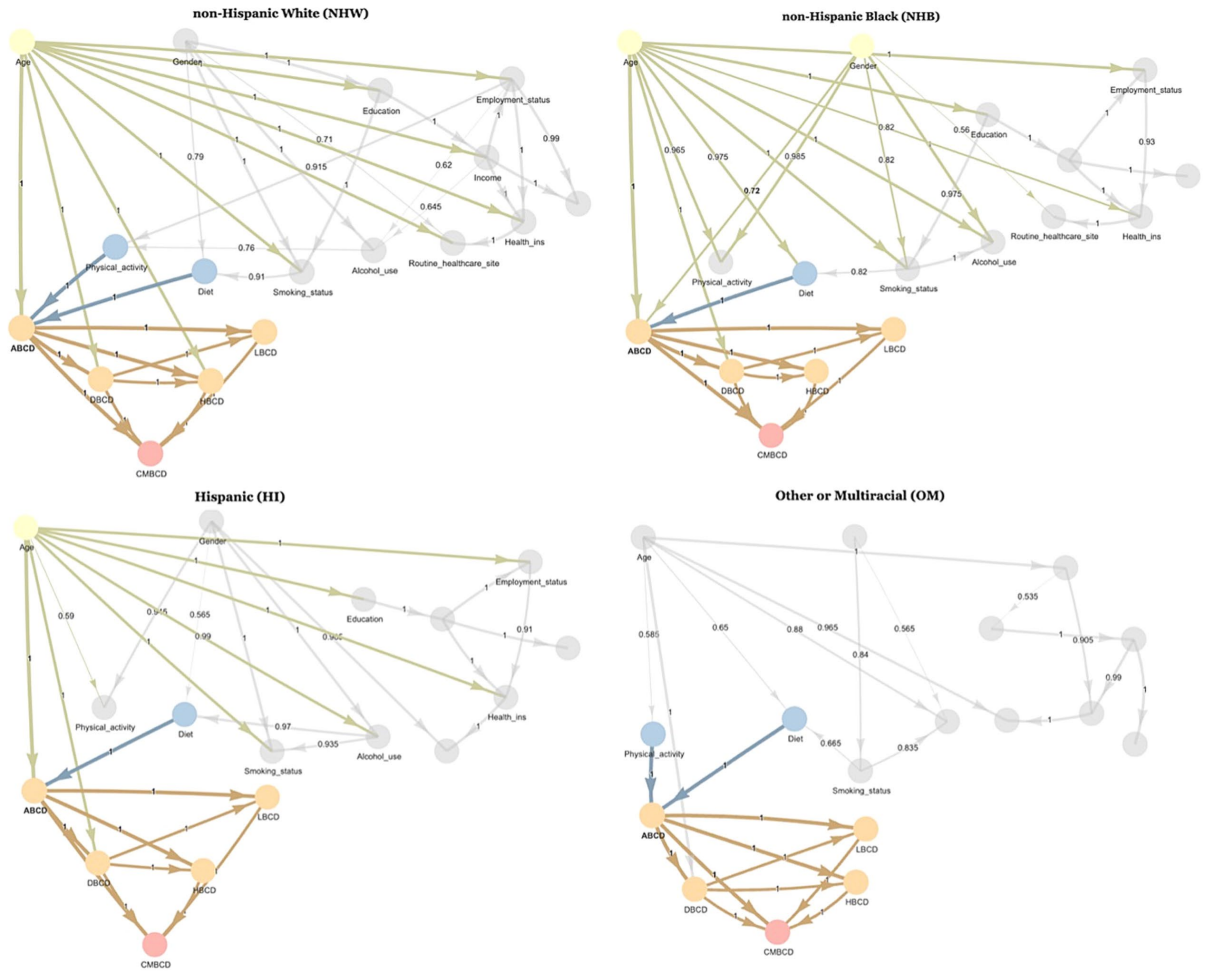
Averaged consensus Bayesian networks learned from NHANES 1999–2018 stratified by ethno-racial group. Highlighted nodes are Markov blanket of ABCD node. Number in middle of arrow and thickness of arrow indicate proportion of bootstrapped samples which included that relationship. ABCD, adiposity-based chronic disease; DBCD, dysglycemia-based chronic disease; HBCD, hypertension-based chronic disease; LBCD, lipid-based chronic disease; CMBCD, cardiometabolic-based chronic disease.

After stratifying the sample based on ethno-racial group, the Bayesian networks learned for each ethno-racial group showed distinctive differences (Figure 3). In the NHW network, the parent nodes of ABCD were age, physical activity, and diet—which were consistent with the findings of the overall network. However, in the NHB network, the parent nodes of ABCD were age, gender, and diet. In the HI network, the parent nodes of ABCD were age and diet. In the OM network, the parent nodes of ABCD were physical activity and diet.

### Network queries by demographics

**Figure 4:**
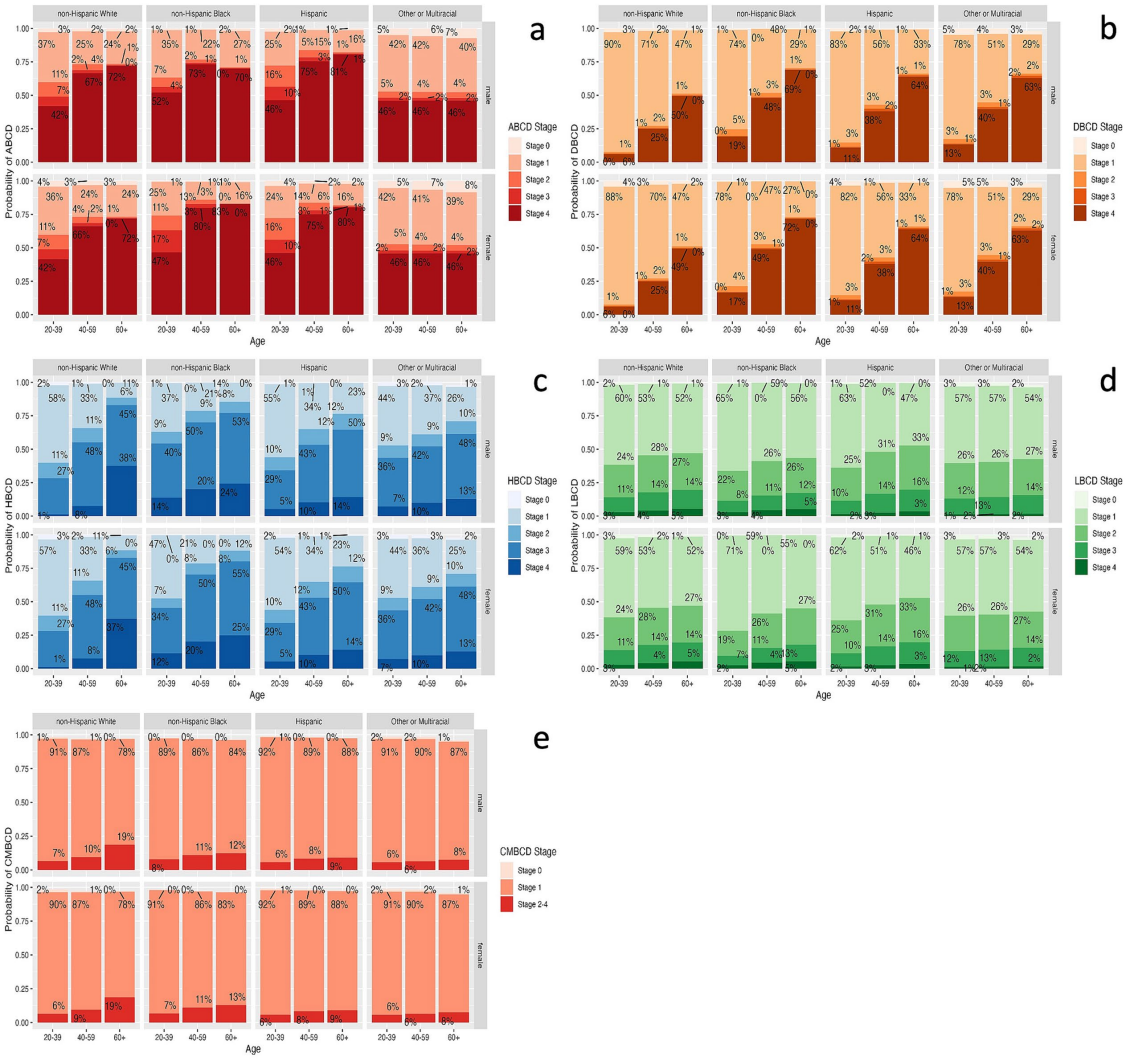
Estimates for prevalence of each stage of ABCD **(a)**, DBCD **(b)**, HBCD (c), LBCD **(d)**, and CMBCD **(e)** for each combination of age, gender, and ethno-racial group based on conditional probability queries of learned Bayesian networks. ABCD, adiposity-based chronic disease; DBCD, dysglycemia-based chronic disease; HBCD, hypertension-based chronic disease; LBCD, lipid-based chronic disease; CMBCD, cardiometabolic-based chronic disease.

Given the learned network structure for each ethno-racial group, conditional probability queries were conducted by instantiating each combination of age, gender, and ethno-racial group and estimating the probability of all stages of ABCD, DBCD, HBCD, LBCD, and CMBCD. In terms of ABCD, the highest probability of stage 4 was among NHB females 60+ years old (83%; Figure 4a). In terms of DBCD, the highest probability of stage 4 was among NHB females 60+ years old (72%; Figure 4b). In terms of HBCD, the highest probability of stage 4 was among NHW males 60+ years old (38%; Figure 4c). In terms of LBCD, the highest probability of stage 4 was among NHB and NHW males and females 60+ years old (5%; Figure 4d). In terms of CMBCD, the highest probability of stage 2–4 was among NHW males and females 60+ years old (19%; Figure 4e).

### Network queries for behavioral factors

**Figure 5:**
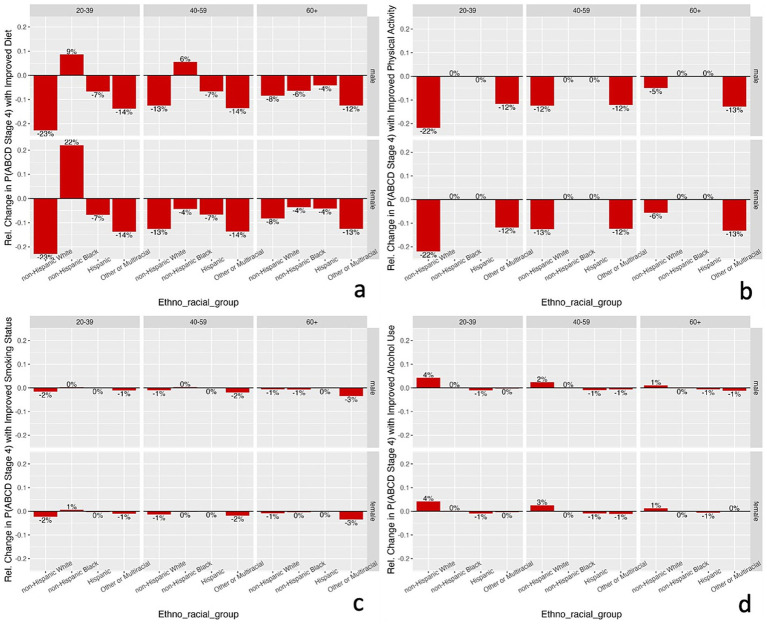
Relative change in probability of ABCD stage 4 with improved diet **(a)**, physical activity **(b)**, smoking status **(c)**, and alcohol use **(d)** for each combination of age, gender, and ethno-racial group based on conditional probability queries of learned Bayesian networks. Improved diet refers to comparing non-DASH-accordant diet to DASH-accordant diet. Improved physical activity refers to comparing none to sufficient physical activity. Improved smoking status refers to comparing current smoker to never smoker. Improved alcohol use refers to comparing heavy to no alcohol use. ABCD, adiposity-based chronic disease; DASH; Dietary Approaches to Stop Hypertension.

Given the learned network structure for each ethno-racial group, conditional probability queries were conducted by instantiating each combination of age, gender, and ethno-racial group along with one of the behavioral factors (diet, physical activity, smoking status, alcohol use) to analyze the impact of changing values of behavioral variables on the cardiometabolic drivers. The impact of improving each behavioral factor on ABCD stage 4 (the most proximal cardiometabolic driver) is shown in Figure 5 (see Supplementary Figures 1–4 for the results for all other drivers). Compared with a non-Dietary Approaches to Stop Hypertension (DASH)-accordant diet, a DASH-accordant diet was associated with the largest decrease in probability of ABCD stage 4 specifically among NHW males and females who were 20–39 years old (23% decrease; Figure 5a). Among NHB males and females 20–39 years old and NHB males 40–59 years old, there was a paradoxical increase in probability of ABCD stage 4 when comparing those with a DASH-accordant diet to those with a non-DASH-accordant diet.

Compared to those with no physical activity, sufficient physical activity was associated with the largest decrease in probability of ABCD stage 4 specifically among NHW males and females who were 20–39 years old (22% decrease; Figure 5b). Given that the learned networks for NHB and HI did not include a direct or indirect pathway linking physical activity and the cardiometabolic outcomes, the conditional probability queries showed no effect of sufficient physical activity on the probability of ABCD stage 4 for individuals in those ethno-racial groups irrespective of age or gender.

Compared to those who were current smokers, never smoking was associated with the largest decrease in probability of ABCD stage 4 among ORM males and females 60+ years old (3% decrease, Figure 5c). Compared to those with heavy alcohol use, no alcohol use was associated with the largest absolute change in probability of ABCD stage 4 among NHW males and females 20–39 years old (4% increase, Figure 5d).

## Discussion

This study utilized 20 years of NHANES data with variables spanning socio-demographics, SDOH, and behavioral factors, as well as staged measures of adiposity, dysglycemia, hypertension, dyslipidemia, and CVD to construct a Bayesian network and analyze the inter-relationships among these variables. Notably, we found several pathways that mediated the indirect association from ethno-racial group to cardiometabolic outcomes; sequentially, these included pathways from (1) ethno-racial group to education and behavioral factors (diet); (2) education to behavioral factors (smoking, physical activity, and – via income – to alcohol); (3) and behavioral factors to ABCD and then the other cardiometabolic drivers. Bayesian networks are intended to represent conditional dependencies among nodes, so the learned edges can only be interpreted to imply causality under specific assumptions, including that there must not be a latent variable that acts as a confounder (15). Additionally, the cross-sectional nature of the NHANES data limits causal interpretation of the findings. However, by incorporating expert knowledge through disallowing edges that are unreasonable (e.g., diet to income) in the model learning process, network interpretability of edges was increased.

Furthermore, individual edges mediating pathways from ethno-racial group to cardiometabolic outcomes were scientifically substantiated based on prior literature. Regarding the ethno-racial group to education pathway, disparities in education by ethno-racial group in the US due to barriers of access have been well-established. Even though these disparities have been decreasing over time, there has been a greater high school dropout rate and lower college enrollment rate in NHB and HI compared to NHW and Asian individuals of the same age (29). Regarding the ethno-racial group to diet pathway, dietary consumption of various nutrients varies by ethno-racial group. For instance, there is higher protein, fruit, and carbohydrate consumption among HI and non-Hispanic Asian individuals compared to NHW and NHB individuals (30). Moreover, non-Hispanic Asian individuals have the highest dietary quality scores as a consequence of higher fish and lower fat intakes (31). Regarding the education to behavioral factors pathway, the highest level of education attained has been found to be inversely related to lifetime CVD risk regardless of socio-economic status (32). Better health literacy, which is positively related to education level, has been consistently associated with better diet and physical activity (33, 34). However, the relationship of education and alcohol use is more complex, with higher educational level associated with reduced daily alcohol consumption and consumption of distilled spirits but greater alcohol intake frequency and consumption of wine (35). Regarding the behavioral factors to cardiometabolic drivers pathway, poor physical activity and diet are well-known contributors to overweight and obesity (ABCD stages 2–4). In addition, these behavioral risk factors have been linked to other cardiometabolic drivers, including T2D, hypertension, and hyperlipidemia (36–38). Given the presence of behavioral factors in pathways relating ethno-racial group and cardiometabolic outcomes, as well as the strong evidence supporting these pathways, interventions should be implemented among NHB and HI to address these behavioral factors—particularly diet and physical activity—to reduce ethno-racial cardiometabolic disparities. However, individual behaviors are strongly influenced by structural and neighborhood determinants of health (3). Therefore, interventions must include population-level efforts to address root cause structural determinants.

In addition to pathways mediating the association between ethno-racial group and cardiometabolic drivers, separate Bayesian networks were learned for each ethno-racial group classified in the NHANES dataset. Physical activity was a parent node of ABCD and, by extension, the other cardiometabolic drivers in the Bayesian networks learned for NHW and OM but not in those learned for NHB or HI. Subsequently, conditional probability queries were conducted using the learned Bayesian networks and revealed differences in the effect of diet and physical activity on the probability of ABCD stage 4 (the most proximal cardiometabolic driver) across different combinations of age, gender, and ethno-racial group. While diet was associated with ABCD in the Bayesian networks learned for each ethno-racial group, improving diet from non-DASH-accordant to DASH-accordant was associated with a larger improvement in ABCD stage 4 for NHW individuals than for NHB and HI individuals. The results of these two analyses suggest that the degree to which behavioral factors—specifically diet and physical activity—influence cardiometabolic outcomes differs by ethno-racial group.

Taken together, the aforementioned findings suggest that, while traditional behavioral factors such as diet and physical activity mediated the pathways from ethno-racial group to cardiometabolic outcomes, they were more influential among NHW individuals than NHB or HI individuals in determining cardiometabolic outcomes. This may reflect other unmeasured underlying factors (or “hidden variables”) that have a stronger relative impact on cardiometabolic drivers among NHB and HI individuals. While individual-level food security, health insurance, and routine healthcare site were included in the network for this study and not found to be linked to the cardiometabolic drivers, the unmeasured factors can include potentially other individual-level (access to healthcare, health literacy, and housing stability) and neighborhood-level (food environment, built environment, and exposure environmental pollutants) structural determinants of health. All of these factors have been shown to disproportionately impact NHB and HI individuals (3, 39–42). Subsequent research should aim to incorporate these neighborhood-level structural determinants into network analyses to understand how they interact with individual demographic and behavioral factors to influence cardiometabolic outcomes.

One paradoxical finding from this analysis was that a DASH-accordant diet was actually associated with a higher probability of ABCD stage 4 among NHB males and females 20–39 years old and NHB males 40–59 years old compared to a non-DASH-accordant diet. This may reflect an unobserved confounding variable unique to this demographic that is associated with better diet and worse cardiometabolic outcomes. Alternatively, this can be an isolated case of reverse causality, whereby young NHB individuals with advanced cardiometabolic drivers are encouraged to pursue a healthier diet. This finding requires further research to clarify the factors underlying this trend. More broadly, this unique type of analysis including conditional queries illustrates the utility of using Bayesian networks to show the interaction of multiple variables in providing specific estimates for each combination of demographic variables. Such research that helps to better understand the impacts of behavioral factors based on specific demographic characteristics can help inform more tailored interventions at an individual or population level.

One of the main strengths of this study was the merging and harmonization of 20 years of NHANES data with variables spanning multiple tiers. Additionally, instead of focusing on a single health outcome, this study included multiple cardiometabolic drivers to enable comprehensive analysis of factors underlying CVD. The focus on Bayesian networks rather than traditional regression analyses facilitated the complex analysis of multi-tiered cardiometabolic variables. Additionally, the comparison of networks of health variables across demographic factors including ethno-racial group was a novel approach that has not been done in previous studies reviewed by the authors. Lastly, conditional probability queries were performed using the Bayesian networks to clarify differential effects of behavioral factors across specific combinations of demographic variables.

Meanwhile, our analysis also had some limitations, including the use of cross-sectional instead of longitudinal data which limits the ability to derive causal conclusions. Given that cardiometabolic conditions are chronic diseases that develop over an extended time-course, future studies should use dynamic Bayesian networks to analyze longitudinal datasets in order to understand the influence of behavioral factors and SDOH at time points predating the incidence of CMBCD. Furthermore, while this study utilized 20 years of NHANES data in order to assure a sufficient sample size for accurate network learning, the integration of a potential time effect was not possible given the cross-sectional nature of the data and the limitations of the Bayesian network methodology. However, the associations derived from the network have been individually reported across time in prior literature. Additionally, comparison of results from the conditional probability queries was based on point estimates as there has not been any validated hypothesis testing approach developed for Bayesian network queries. This analysis also did not incorporate NHANES sampling weights as no validated approach to incorporate sampling weights into Bayesian network learning could be identified. However, to minimize bias introduced by this limitation, only analyses stratified by age, gender, and ethno-racial group were conducted. Lastly, this analysis only included individual-level social determinants and not neighborhood-level factors, which influence individual behaviors and health outcomes. Future analyses should integrate geospatial data from neighborhood social determinants.

In sum, the association between ethno-racial group and cardiometabolic drivers was mediated through pathways that involve education, income, and behavioral factors. However, traditional risk factors – mainly diet and physical activity—were more influential among NHW individuals than NHB or HI individuals in determining cardiometabolic outcomes, which may result from unmeasured individual- and neighborhood-level structural determinants having a stronger relative impact on cardiometabolic drivers in these demographics. Finally, Bayesian networks provided a unique approach to analyze the complex interaction of multi-tiered variables in determining cardiometabolic outcomes and conduct queries to estimate the probability of a target node given specific combinations of values of other variables in the network.

## Data Availability

Publicly available datasets were analyzed in this study. This data can be found here: www.cdc.gov/nchs/nhanes/index.htm.
