# Supplementary material for: Bayesian network model of ethno-racial disparities in cardiometabolic-based chronic disease using NHANES 1999–2018
Source: Front Public Health. 2024 Oct 15;12:1409731. doi: 10.3389/fpubh.2024.1409731 (PMC11519814; doi:10.3389/fpubh.2024.1409731)
Supplement: Supplementary file 1 [file Data_Sheet_1.DOCX]

**Bayesian network model of ethno-racial disparities in cardiometabolic-based chronic disease using NHANES 1999-2018**

*Masih A Babagoli^1*^, Michael J Beller^2^, Juan P Gonzalez-Rivas^3,4,5^, Ramfis Nieto-Martinez^3,4,6^, Faris Gulamali^1^, Jeffrey I Mechanick^7,8^*

^1^ Icahn School of Medicine at Mount Sinai, New York, New York, USA

^2^ Beller Tech LLC, New York, New York, USA

^3^ Department of Global Health and Population, Harvard TH Chan School of Public Health, Boston, Massachusetts, USA

^4^ Foundation for Clinic, Public Health, and Epidemiology Research in Venezuela (FISPEVEN INC), Caracas, Venezuela

^5^ International Clinical Research Center (ICRC), St. Ann's University Hospital, Brno, Czech Republic

^6^ Precision Care Clinic Corp., Saint Cloud, Florida, USA

^7^ The Marie-Josée and Henry R. Kravis Center for Cardiovascular Health at Mount Sinai Fuster Heart Hospital, New York, New York, USA

^8^ Division of Endocrinology, Diabetes and Bone Disease, Icahn School of Medicine at Mount Sinai, New York, New York, USA

* Corresponding author: masih.babagoli@icahn.mssm.edu

Supplementary Table 1: Definitions of all model variables included in the Bayesian network based on available data from NHANES.

| **Tier** | **Model variable** | **Values** | **Notes** |
| --- | --- | --- | --- |
| Non-modifiable socio-demographics | Age | 20-39, 40-59, 60+ years old | Relevant NHANES variables: RIDAGEYR. |
|  | Gender | Male, female | Relevant NHANES variables: RIAGENDR. |
|  | Ethno-racial group | Hispanic, non-Hispanic White, non-Hispanic Black, other race or multiracial | Relevant NHANES variables: RIDRETH1. |
| Modifiable socio-demographics | Education | Less than high school, high school/GED, some college/AA degree, college graduate or more | Relevant NHANES variables: DMDEDUC2. |
|  | Income | <100% FPL, 100-199% FPL, 200-399% FPL, >400% FPL | Relevant NHANES variables: INDFMPIR. |
|  | Employment status | Employed/homemaker/student, retired, unemployed/unable to work | Relevant NHANES variables: OCQ380, OCD150 (2001-2018), OCQ150 (1999-2000). |
| Social determinants of health | Household food security | Full food security, marginal food security, low food security, very low food security | Based on US Food Security Survey Module.^1^ Relevant NHANES variables: FSDHH (2003-2018), HHFDSEC (1999-2002). |
|  | Health insurance | No health insurance, private health insurance, public health insurance (Medicare), public health insurance (other) | Relevant NHANES variables: HIQ031 (2005-2018), HID030 (1999-2004). |
|  | Routine healthcare site | No routine healthcare site, emergency department, site other than emergency department | Relevant NHANES variables: HUQ030, HUQ041 (2013-2018), HUQ040 (1999-2012). |
| Behavioral factors | Diet | DASH-accordant, not DASH-accordant | Defined by accordance to the DASH diet based on scoring criteria by Mellen et al.^2^ Relevant NHANES variables: DR1xxx (2003-2018), DR2 (2003-2018), DRXxxx (1999-2002). |
|  | Physical activity | None, not sufficient, sufficient | Defined by minutes per week of moderate physical activity or equivalent (0, 1-150, ≥150).^3,4^ Relevant NHANES variables: see variables under PAQ section. For 1999-2006 where full GPAQ not available, summed minutes/week walk or bicycle, household tasks, and leisure time activity. |
|  | Alcohol use | None, light, moderate, heavy | Defined by drinks per week (0, <1, 1-8, or ≥8 respectively).^5^ Relevant NHANES variables: ALQ121 (2017-2018), ALQ130 (2017-2018), ALQ111 (2017-2018), ALQ121 (2017-2018), ALQ120Q/U (1999-2016), ALQ130 (1999-2016), ALQ110 (1999-2016). |
|  | Smoking status | Never, former, current | Defined as Current smoker if currently smokes or quit within the past one year. Relevant NHANES variables: SMQ020, SMQ040, SMQ050Q/U. |
| Secondary cardiometabolic drivers | ABCD | Stage 0, Stage 1, Stage 2, Stage 3, Stage 4 | Stage 0: (DASH-accordant diet AND sufficient physical activity) AND normal/underweight BMI  Stage 1: (not DASH-accordant diet OR none/not sufficient physical activity) AND normal/underweight BMI  Stage 2: Overweight BMI AND (absent CVD, absent prediabetes/diabetes, absent prehypertension/hypertension, total cholesterol<200)  Stage 3: Obese BMI AND (absent CVD, absent prediabetes/diabetes, absent prehypertension/hypertension, total cholesterol<200)  Stage 4: Overweight/obese BMI AND (present CVD, present prediabetes/diabetes, present prehypertension/hypertension, total cholesterol≥200)  Relevant NHANES variables: BMXBMI, DIQ010, DIQ050, DIQ070, LBXGH, BPXSY1/2/3, BPXDI1/2/3, BPQ040A, BPQ020, LBXTC, MCQ160B/C/E/F (in addition to previously defined model variables). |
|  | DBCD | Stage 0, Stage 1, Stage 2, Stage 3, Stage 4 | Stage 0: (DASH-accordant diet AND sufficient physical activity AND normal/underweight BMI) AND absent prediabetes/diabetes  Stage 1: (not DASH-accordant diet OR none/not sufficient physical activity OR overweight/obese BMI) AND absent prediabetes/diabetes  Stage 2: Prediabetes AND (absent CVD, absent renal disease, absent retinopathy, absent prehypertension/hypertension, total cholesterol<200)  Stage 3: Diabetes AND (absent CVD, absent renal disease, absent retinopathy, absent prehypertension/hypertension, total cholesterol<200)  Stage 4: Prediabetes/diabetes AND (present CVD, present renal disease, present retinopathy, present prehypertension/hypertension, total cholesterol≥200)  Relevant NHANES variables: BMXBMI, DIQ010, DIQ050, DIQ070, LBXGH, BPXSY1/2/3, BPXDI1/2/3, BPQ040A, BPQ020, LBXTC, MCQ160B/C/E/F, DIQ080, LBXSCR, KIQ022 (2001-2018), KIQ020 (1999-2000) (in addition to previously defined model variables). |
|  | HBCD | Stage 0, Stage 1, Stage 2, Stage 3, Stage 4 | Stage 0: (DASH-accordant diet AND sufficient physical activity AND normal/underweight BMI AND absent prediabetes/diabetes) AND absent prehypertension/hypertension  Stage 1: (not DASH-accordant diet OR none/not sufficient physical activity OR overweight/obese BMI OR present prediabetes/diabetes) AND absent prehypertension/hypertension  Stage 2: Prehypertension AND (absent CVD AND absent renal disease)  Stage 3: Hypertension AND (absent CVD AND absent renal disease)  Stage 4: Prehypertension/hypertension AND (present CVD OR present renal disease)  Relevant NHANES variables: BMXBMI, DIQ010, DIQ050, DIQ070, LBXGH, BPXSY1/2/3, BPXDI1/2/3, BPQ040A, BPQ020, MCQ160B/C/E/F, LBXSCR, KIQ022 (2001-2018), KIQ020 (1999-2000) (in addition to previously defined model variables). |
|  | LBCD | Stage 0, Stage 1, Stage 2, Stage 3, Stage 4 | Stage 0: (DASH-accordant diet AND sufficient physical activity AND normal/underweight BMI AND absent prediabetes/diabetes) AND total cholesterol<200  Stage 1: (not DASH-accordant diet OR none/not sufficient physical activity OR overweight/obese BMI OR present prediabetes/diabetes) AND total cholesterol<200  Stage 2: Total cholesterol 200-239 AND (absent CVD)  Stage 3: Total cholesterol≥240 AND (absent CVD)  Stage 4: Total cholesterol≥200 AND (present CVD)  Relevant NHANES variables: BMXBMI, DIQ010, DIQ050, DIQ070, LBXGH, LBXTC, MCQ160B/C/E/F (in addition to previously defined model variables). |
| Cardiovascular disease | CMBCD | Stage 0, Stage 1, Stage 2-4 | Stage 0: absent CVD AND (ABCD Stage 0, DBCD Stage 0, HBCD Stage 0, AND LBCD Stage 0)  Stage 1: absent CVD AND (ABCD Stage 1-4, DBCD Stage 1-4, HBCD Stage 1-4, OR LBCD Stage 1-4)  Stage 2-4: present CVD AND (ABCD Stage 1-4, DBCD Stage 1-4, HBCD Stage 1-4, OR LBCD Stage 1-4)  Relevant NHANES variables: MCQ160B/C/E/F (in addition to previously defined model variables). |

Abbreviations: Dietary Approaches to Stop Hypertension (DASH), adiposity-based chronic disease (ABCD), dysglycemia-based chronic disease (DBCD), hypertension-based chronic disease (HBCD), and lipid-based chronic disease (LBCD), cardiometabolic-based chronic disease (CMBCD), cardiovascular disease (CVD), body mass index (BMI), Global Physical Activity Questionnaire (GPAQ).

Supplementary Table 2a: Relationships/edges not allowed (“blacklisted”) based on *a priori* knowledge in the structure learning of Bayesian networks.

| **From** | **To** |
| --- | --- |
| Gender | Age |
| Ethno_racial_group | Age |
| Age | Gender |
| Ethno_racial_group | Gender |
| Age | Ethno_racial_group |
| Gender | Ethno_racial_group |
| Education | Age |
| Income | Age |
| Employment_status | Age |
| HH_food_security | Age |
| Health_ins | Age |
| Routine_healthcare_site | Age |
| Alcohol_use | Age |
| Smoking_status | Age |
| Physical_activity | Age |
| Diet | Age |
| ABCD | Age |
| DBCD | Age |
| HBCD | Age |
| LBCD | Age |
| CMBCD | Age |
| Education | Gender |
| Income | Gender |
| Employment_status | Gender |
| HH_food_security | Gender |
| Health_ins | Gender |
| Routine_healthcare_site | Gender |
| Alcohol_use | Gender |
| Smoking_status | Gender |
| Physical_activity | Gender |
| Diet | Gender |
| ABCD | Gender |
| DBCD | Gender |
| HBCD | Gender |
| LBCD | Gender |
| CMBCD | Gender |
| Education | Ethno_racial_group |
| Income | Ethno_racial_group |
| Employment_status | Ethno_racial_group |
| HH_food_security | Ethno_racial_group |
| Health_ins | Ethno_racial_group |
| Routine_healthcare_site | Ethno_racial_group |
| Alcohol_use | Ethno_racial_group |
| Smoking_status | Ethno_racial_group |
| Physical_activity | Ethno_racial_group |
| Diet | Ethno_racial_group |
| ABCD | Ethno_racial_group |
| DBCD | Ethno_racial_group |
| HBCD | Ethno_racial_group |
| LBCD | Ethno_racial_group |
| CMBCD | Ethno_racial_group |
| HH_food_security | Education |
| Health_ins | Education |
| Routine_healthcare_site | Education |
| Alcohol_use | Education |
| Smoking_status | Education |
| Physical_activity | Education |
| Diet | Education |
| ABCD | Education |
| DBCD | Education |
| HBCD | Education |
| LBCD | Education |
| CMBCD | Education |
| HH_food_security | Income |
| Health_ins | Income |
| Routine_healthcare_site | Income |
| Alcohol_use | Income |
| Smoking_status | Income |
| Physical_activity | Income |
| Diet | Income |
| ABCD | Income |
| DBCD | Income |
| HBCD | Income |
| LBCD | Income |
| CMBCD | Income |
| HH_food_security | Employment_status |
| Health_ins | Employment_status |
| Routine_healthcare_site | Employment_status |
| Alcohol_use | Employment_status |
| Smoking_status | Employment_status |
| Physical_activity | Employment_status |
| Diet | Employment_status |
| ABCD | Employment_status |
| DBCD | Employment_status |
| HBCD | Employment_status |
| LBCD | Employment_status |
| CMBCD | Employment_status |
| Alcohol_use | HH_food_security |
| Smoking_status | HH_food_security |
| Physical_activity | HH_food_security |
| Diet | HH_food_security |
| ABCD | HH_food_security |
| DBCD | HH_food_security |
| HBCD | HH_food_security |
| LBCD | HH_food_security |
| CMBCD | HH_food_security |
| Alcohol_use | Health_ins |
| Smoking_status | Health_ins |
| Physical_activity | Health_ins |
| Diet | Health_ins |
| ABCD | Health_ins |
| DBCD | Health_ins |
| HBCD | Health_ins |
| LBCD | Health_ins |
| CMBCD | Health_ins |
| Alcohol_use | Routine_healthcare_site |
| Smoking_status | Routine_healthcare_site |
| Physical_activity | Routine_healthcare_site |
| Diet | Routine_healthcare_site |
| ABCD | Routine_healthcare_site |
| DBCD | Routine_healthcare_site |
| HBCD | Routine_healthcare_site |
| LBCD | Routine_healthcare_site |
| CMBCD | Routine_healthcare_site |
| ABCD | Alcohol_use |
| DBCD | Alcohol_use |
| HBCD | Alcohol_use |
| LBCD | Alcohol_use |
| CMBCD | Alcohol_use |
| ABCD | Smoking_status |
| DBCD | Smoking_status |
| HBCD | Smoking_status |
| LBCD | Smoking_status |
| CMBCD | Smoking_status |
| ABCD | Physical_activity |
| DBCD | Physical_activity |
| HBCD | Physical_activity |
| LBCD | Physical_activity |
| CMBCD | Physical_activity |
| ABCD | Diet |
| DBCD | Diet |
| HBCD | Diet |
| LBCD | Diet |
| CMBCD | Diet |
| CMBCD | ABCD |
| CMBCD | DBCD |
| CMBCD | HBCD |
| CMBCD | LBCD |
| LBCD | ABCD |
| LBCD | DBCD |
| HBCD | ABCD |
| HBCD | DBCD |
| DBCD | ABCD |
| Ethno_racial_group | ABCD |
| Ethno_racial_group | DBCD |
| Ethno_racial_group | HBCD |
| Ethno_racial_group | LBCD |
| Ethno_racial_group | CMBCD |

Abbreviations: adiposity-based chronic disease (ABCD), dysglycemia-based chronic disease (DBCD), hypertension-based chronic disease (HBCD), and lipid-based chronic disease (LBCD), cardiometabolic-based chronic disease (CMBCD)

Supplementary Table 2b: Relationships/edges enforced (“whitelisted”) based on *a priori* knowledge in the structure learning of Bayesian networks.

| **From** | **To** |
| --- | --- |
| ABCD | DBCD |
| ABCD | HBCD |
| ABCD | LBCD |
| DBCD | HBCD |
| DBCD | LBCD |
| ABCD | CMBCD |
| DBCD | CMBCD |
| HBCD | CMBCD |
| LBCD | CMBCD |

Abbreviations: adiposity-based chronic disease (ABCD), dysglycemia-based chronic disease (DBCD), hypertension-based chronic disease (HBCD), and lipid-based chronic disease (LBCD), cardiometabolic-based chronic disease (CMBCD). Note: These relationships reflect considerable epidemiological and mechanistic data that support that adiposity impels dysglycemia, hypertension, and dyslipidemia; dysglycemia impels hypertension and dyslipidemia; and all four drivers impel cardiovascular disease.^6-14^


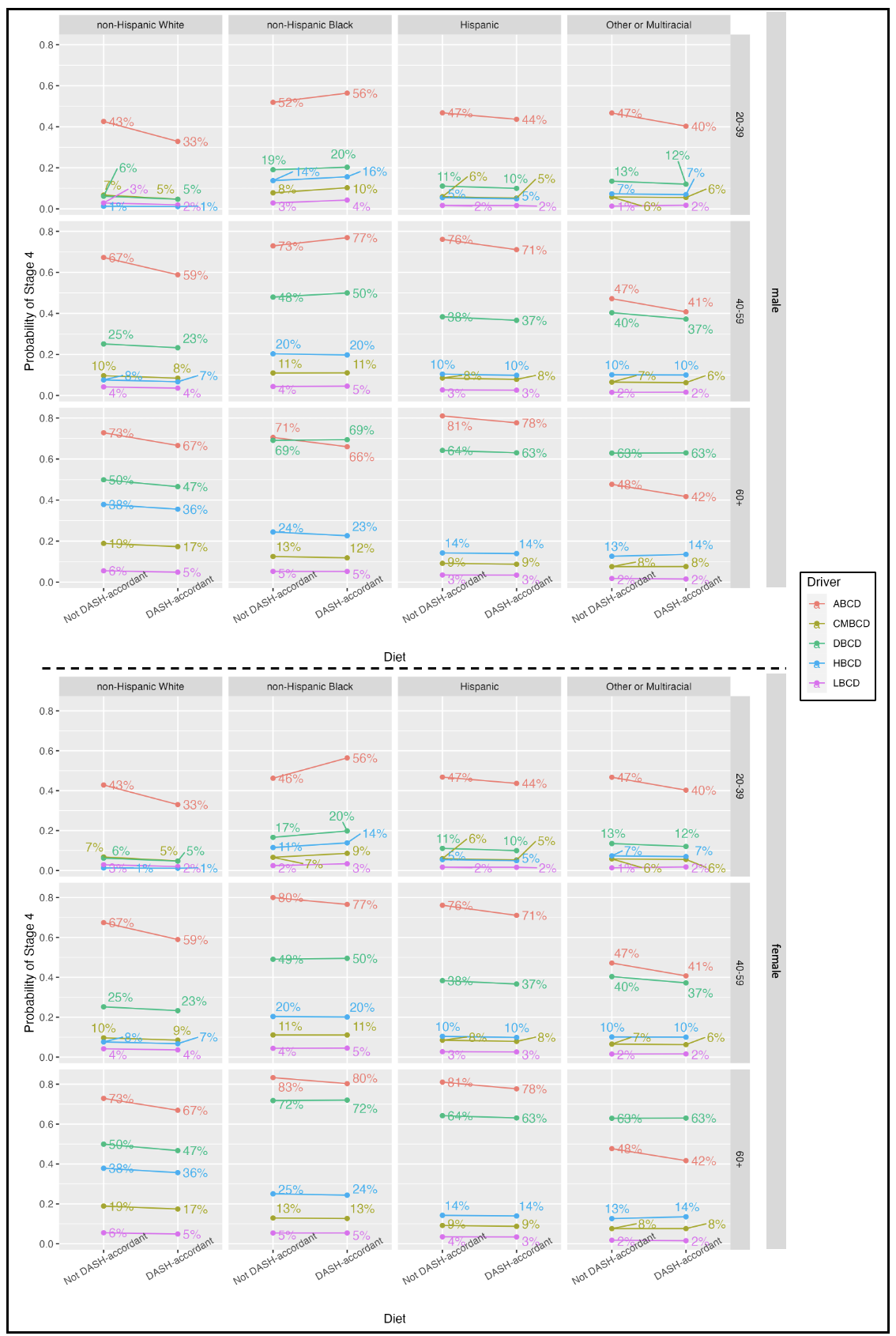


Supplementary Figure 1: Conditional probability queries estimating the probability of all drivers at stage 4 by diet (x-axis), ethno-racial group (columns), age (rows), and gender (top-male, bottom-female). Abbreviations: adiposity-based chronic disease (ABCD), dysglycemia-based chronic disease (DBCD), hypertension-based chronic disease (HBCD), lipid-based chronic disease (LBCD), cardiometabolic-based chronic disease (CMBCD).


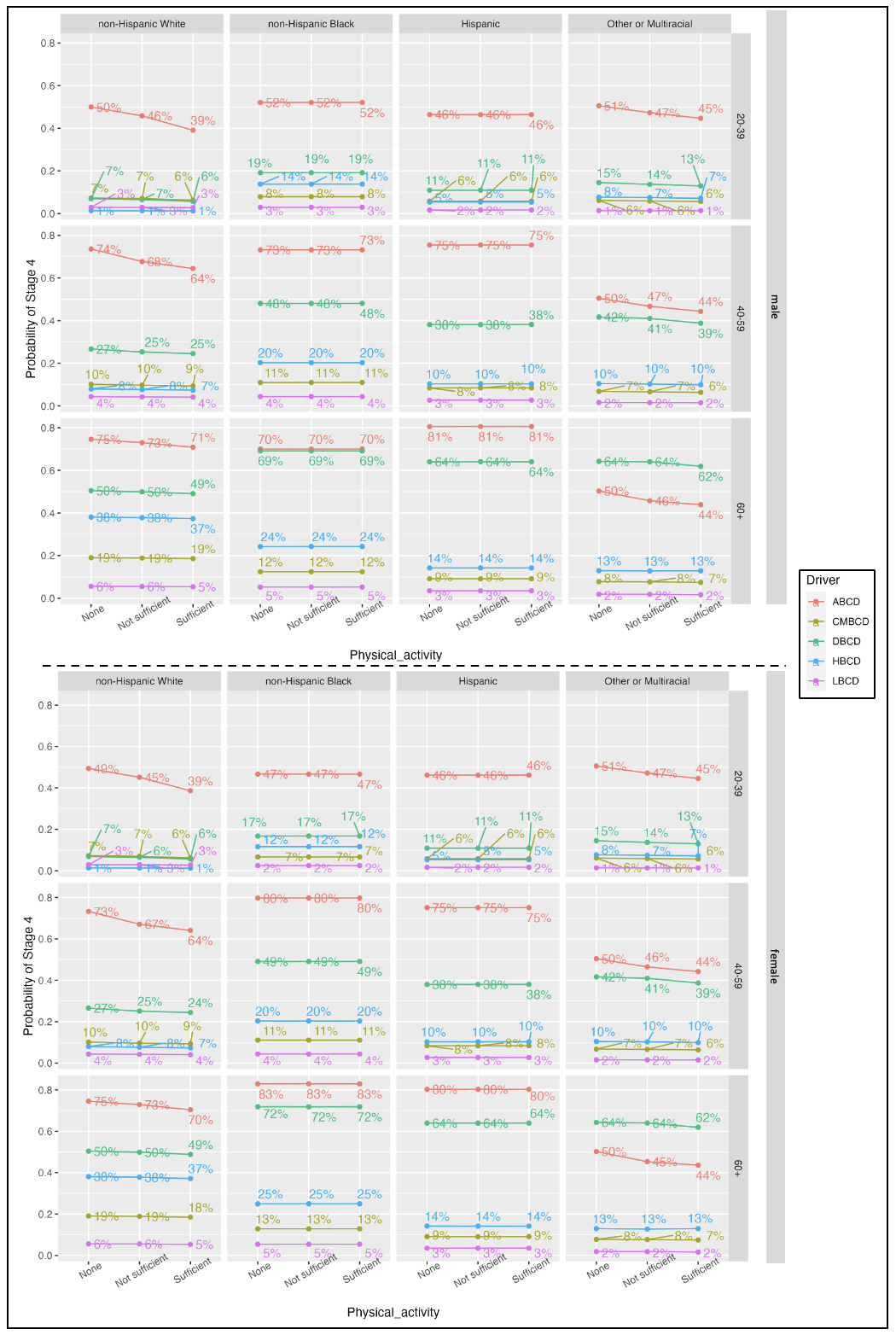


Supplementary Figure 2: Conditional probability queries estimating the probability of all drivers stage 4 by physical activity (x-axis), ethno-racial group (columns), age (rows), and gender (top-male, bottom-female). Abbreviations: adiposity-based chronic disease (ABCD), dysglycemia-based chronic disease (DBCD), hypertension-based chronic disease (HBCD), lipid-based chronic disease (LBCD), cardiometabolic-based chronic disease (CMBCD).


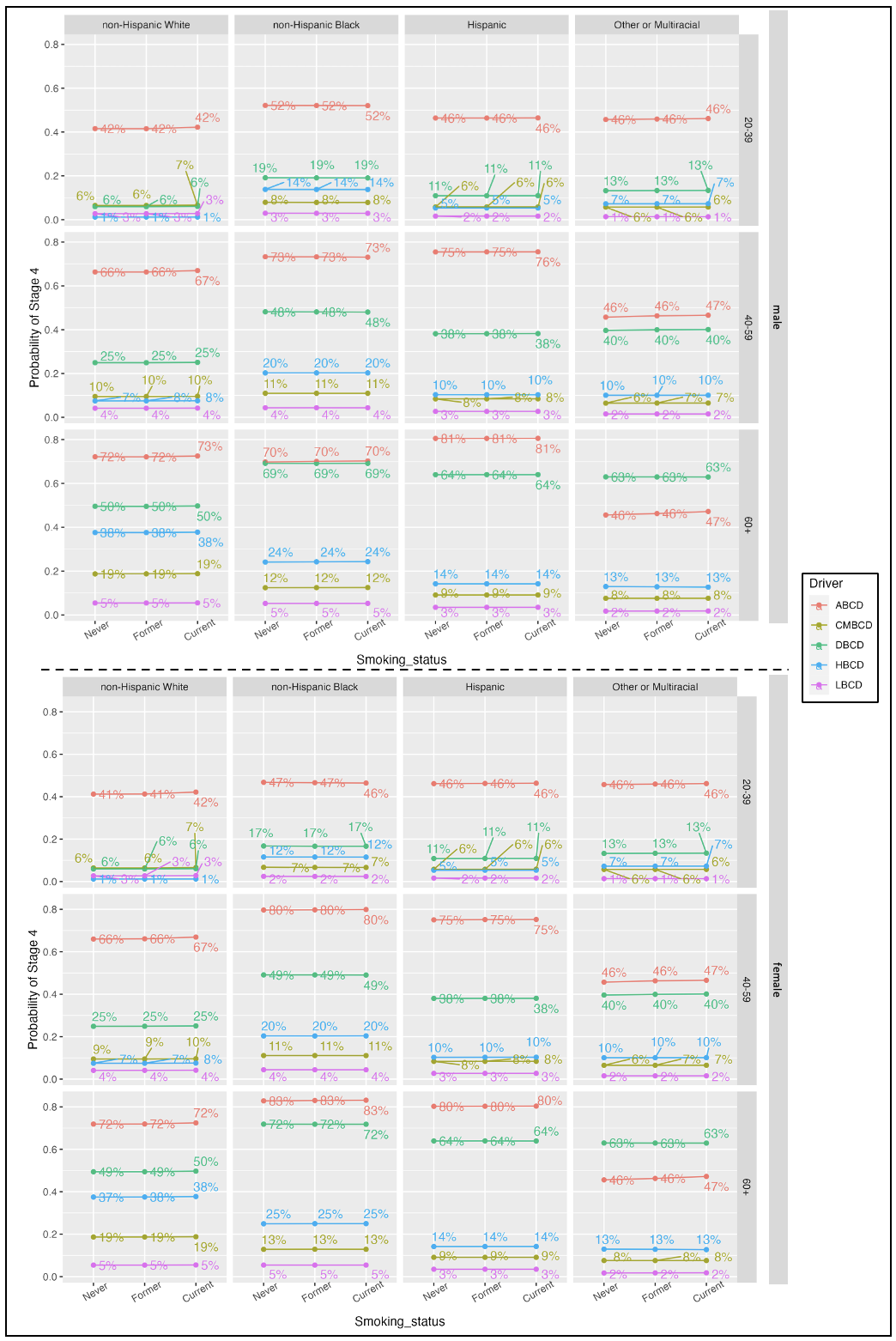


Supplementary Figure 3: Conditional probability queries estimating the probability of all drivers stage 4 by smoking status (x-axis), ethno-racial group (columns), age (rows), and gender (top-male, bottom-female). Abbreviations: adiposity-based chronic disease (ABCD), dysglycemia-based chronic disease (DBCD), hypertension-based chronic disease (HBCD), lipid-based chronic disease (LBCD), cardiometabolic-based chronic disease (CMBCD).


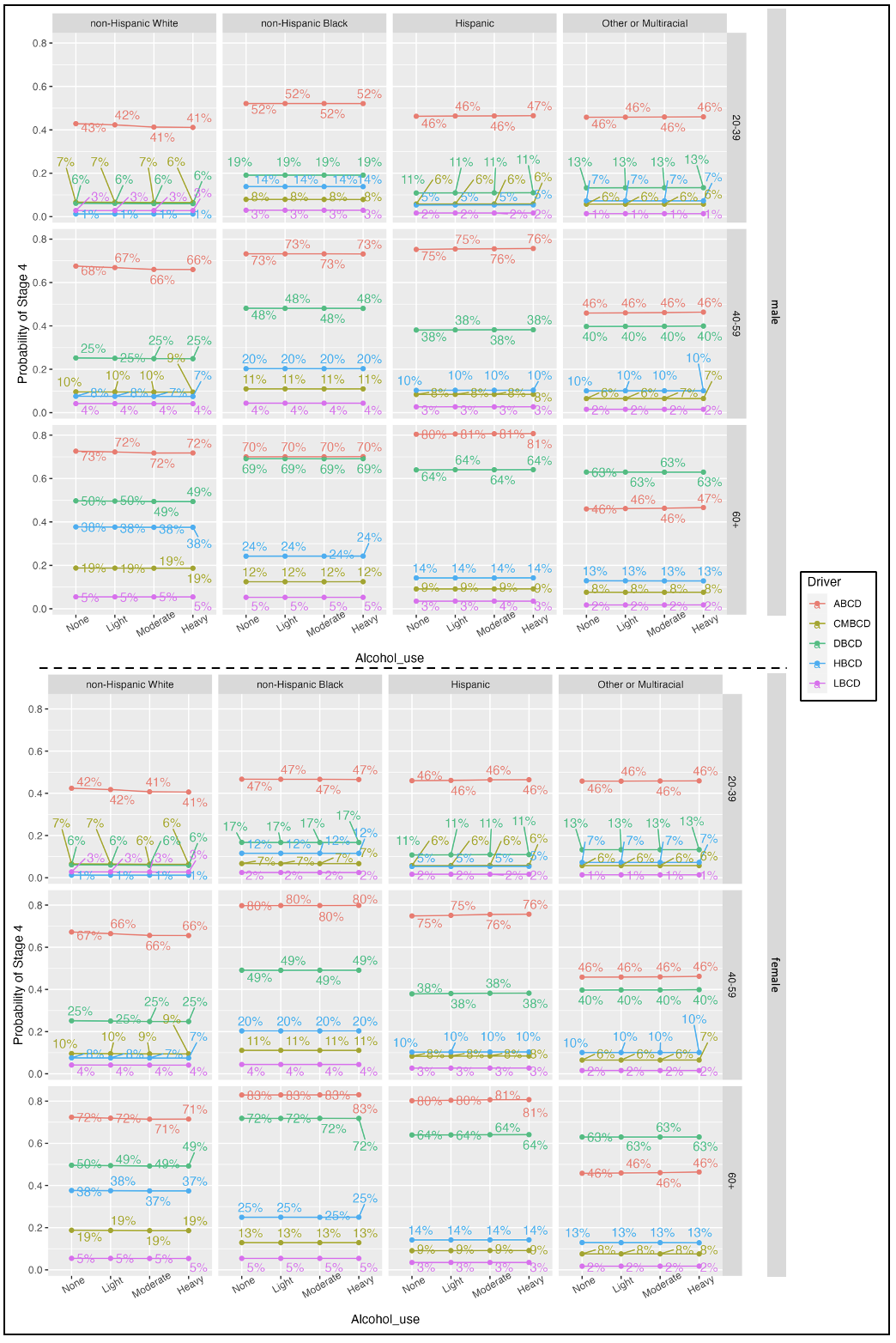


Supplementary Figure 4: Conditional probability queries estimating the probability of all drivers stage 4 by alcohol use (x-axis), ethno-racial group (columns), age (rows), and gender (top-male, bottom-female). Abbreviations: adiposity-based chronic disease *(*ABCD*)*, dysglycemia-based chronic disease *(*DBCD*)*, hypertension-based chronic disease (HBCD), lipid-based chronic disease (LBCD), cardiometabolic-based chronic disease (CMBCD).

R code used for Bayesian network learning and analysis

**#Learning Bayesian network from composite sample**

**##Defining whitelists and blacklists for network**

bl_tiers_02 <- tiers2blacklist(list(

c("Age", "Gender", "Ethno_racial_group"),

c("Education", "Income","Employment_status"),

c("HH_food_security","Health_ins","Routine_healthcare_site"),

c("Alcohol_use","Smoking_status","Physical_activity","Diet"),

c("ABCD","DBCD","HBCD","LBCD"),

c("CMBCD")

))

bl_base_02 <- set2blacklist(c("Age", "Gender", "Ethno_racial_group"))

bl_drivers_02 <- data.frame(from=c("LBCD","LBCD","HBCD","HBCD","DBCD"),to=c("ABCD","DBCD","ABCD","DBCD","ABCD"))

bl_demo_02 <- data.frame(from=c("Ethno_racial_group","Ethno_racial_group","Ethno_racial_group","Ethno_racial_group","Ethno_racial_group"),to=c("ABCD","DBCD","HBCD","LBCD","CMBCD"))

bl_02 <- rbind(bl_base_02, bl_tiers_02,bl_drivers_02,bl_demo_02)

wl_02 <- data.frame(from=c("ABCD","ABCD","ABCD","DBCD","DBCD","ABCD","DBCD","HBCD","LBCD"),to=c("DBCD","HBCD","LBCD","HBCD","LBCD","CMBCD","CMBCD","CMBCD","CMBCD"))

**##Limiting analyses for cases with no missing variables**

model_variables_02<-model_variables_02[complete.cases(model_variables_02),]

**##Learning network structure using hill-climbing algorithm**

dag_02_A<-hc(model_variables_02,blacklist = bl_02,whitelist = wl_02)

undirected.arcs(dag_02_A)

dag_02_A_cpdag<-cpdag(dag_02_A,wlbl = TRUE)

**##Learning network structure using hill-climbing algorithm with 200 bootstrap samples averaged**

dag_02_B<-boot.strength(model_variables_02,R=200,algorithm = "hc",algorithm.args = list(blacklist=bl_02,whitelist=wl_02))

avg_dag_02_B<-averaged.network(dag_02_B)

plot(dag_02_B)

undirected.arcs(avg_dag_02_B)

avg_dag_02_B_cpdag<-cpdag(avg_dag_02_B,wlbl = TRUE)

**##Fitting model parameters using NHANES dataset**

model_02_fitted<-bn.fit(avg_dag_02_B,model_variables_02)

**#Learning Bayesian networks for each ethno-racial group**

**##Defining whitelists and blacklists for network**

bl_tiers_02_byEthno_racial_group <- tiers2blacklist(list(

c("Age", "Gender"),

c("Education", "Income","Employment_status"),

c("HH_food_security","Health_ins","Routine_healthcare_site"),

c("Alcohol_use","Smoking_status","Physical_activity","Diet"),

c("ABCD","DBCD","HBCD","LBCD"),

c("CMBCD")

))

bl_base_02_byEthno_racial_group <- set2blacklist(c("Age", "Gender"))

bl_drivers_02_byEthno_racial_group <- data.frame(from=c("LBCD","LBCD","HBCD","HBCD","DBCD"),to=c("ABCD","DBCD","ABCD","DBCD","ABCD"))

bl_02_byEthno_racial_group <- rbind(bl_base_02_byEthno_racial_group, bl_tiers_02_byEthno_racial_group,bl_drivers_02_byEthno_racial_group)

wl_02_byEthno_racial_group <- data.frame(from=c("ABCD","ABCD","ABCD","DBCD","DBCD","ABCD","DBCD","HBCD","LBCD"),to=c("DBCD","HBCD","LBCD","HBCD","LBCD","CMBCD","CMBCD","CMBCD","CMBCD"))

**##For non-Hispanic White (NHW): Learning network structure using hill-climbing algorithm with 200 bootstrap samples averaged AND fitting model parameters using NHANES dataset**

model_variables_02_NHW<-model_variables_02_byEthno_racial_group %>%

filter(Ethno_racial_group=="non-Hispanic White") %>%

select(-one_of("Ethno_racial_group"))

model_variables_02_NHW<-model_variables_02_NHW[complete.cases(model_variables_02_NHW),]

dag_02_A_NHW<-hc(model_variables_02_NHW,blacklist = bl_02_byEthno_racial_group,whitelist = wl_02_byEthno_racial_group)

graphviz.plot(dag_02_A_NHW)

undirected.arcs(dag_02_A_NHW)

dag_02_A_NHW_cpdag<-cpdag(dag_02_A_NHW,wlbl = TRUE)

graphviz.plot(dag_02_A_NHW_cpdag)

dag_02_B_NHW<-boot.strength(model_variables_02_NHW,R=200,algorithm = "hc",algorithm.args = list(blacklist=bl_02_byEthno_racial_group,whitelist=wl_02_byEthno_racial_group))

avg_dag_02_B_NHW<-averaged.network(dag_02_B_NHW)

strength.plot(avg_dag_02_B_NHW,dag_02_B_NHW)

plot(dag_02_B_NHW)

model_02_NHW_fitted<-bn.fit(avg_dag_02_B_NHW,model_variables_02_NHW)

**##For non-Hispanic Black (NHB): Learning network structure using hill-climbing algorithm with 200 bootstrap samples averaged AND fitting model parameters using NHANES dataset**

model_variables_02_NHB<-model_variables_02_byEthno_racial_group %>%

filter(Ethno_racial_group=="non-Hispanic Black") %>%

select(-one_of("Ethno_racial_group"))

model_variables_02_NHB<-model_variables_02_NHB[complete.cases(model_variables_02_NHB),]

dag_02_A_NHB<-hc(model_variables_02_NHB,blacklist = bl_02_byEthno_racial_group,whitelist = wl_02_byEthno_racial_group)

graphviz.plot(dag_02_A_NHB)

undirected.arcs(dag_02_A_NHB)

dag_02_A_NHB_cpdag<-cpdag(dag_02_A_NHB,wlbl = TRUE)

graphviz.plot(dag_02_A_NHB_cpdag)

dag_02_B_NHB<-boot.strength(model_variables_02_NHB,R=200,algorithm = "hc",algorithm.args = list(blacklist=bl_02_byEthno_racial_group,whitelist=wl_02_byEthno_racial_group))

avg_dag_02_B_NHB<-averaged.network(dag_02_B_NHB)

strength.plot(avg_dag_02_B_NHB,dag_02_B_NHB)

plot(dag_02_B_NHB)

model_02_NHB_fitted<-bn.fit(avg_dag_02_B_NHB,model_variables_02_NHB)

**##For Hispanic (HI): Learning network structure using hill-climbing algorithm with 200 bootstrap samples averaged AND fitting model parameters using NHANES dataset**

model_variables_02_HI<-model_variables_02_byEthno_racial_group %>%

filter(Ethno_racial_group=="Hispanic") %>%

select(-one_of("Ethno_racial_group"))

model_variables_02_HI<-model_variables_02_HI[complete.cases(model_variables_02_HI),]

dag_02_A_HI<-hc(model_variables_02_HI,blacklist = bl_02_byEthno_racial_group,whitelist = wl_02_byEthno_racial_group)

graphviz.plot(dag_02_A_HI)

undirected.arcs(dag_02_A_HI)

dag_02_A_HI_cpdag<-cpdag(dag_02_A_HI,wlbl = TRUE)

graphviz.plot(dag_02_A_HI_cpdag)

dag_02_B_HI<-boot.strength(model_variables_02_HI,R=200,algorithm = "hc",algorithm.args = list(blacklist=bl_02_byEthno_racial_group,whitelist=wl_02_byEthno_racial_group))

avg_dag_02_B_HI<-averaged.network(dag_02_B_HI)

strength.plot(avg_dag_02_B_HI,dag_02_B_HI)

plot(dag_02_B_HI)

model_02_HI_fitted<-bn.fit(avg_dag_02_B_HI,model_variables_02_HI)

**##For Other Race or Multiracial (ORM): Learning network structure using hill-climbing algorithm with 200 bootstrap samples averaged AND fitting model parameters using NHANES dataset**

model_variables_02_ORM<-model_variables_02_byEthno_racial_group %>%

filter(Ethno_racial_group=="Other or Multiracial") %>%

select(-one_of("Ethno_racial_group"))

model_variables_02_ORM<-model_variables_02_ORM[complete.cases(model_variables_02_ORM),]

dag_02_A_ORM<-hc(model_variables_02_ORM,blacklist = bl_02_byEthno_racial_group,whitelist = wl_02_byEthno_racial_group)

graphviz.plot(dag_02_A_ORM)

undirected.arcs(dag_02_A_ORM)

dag_02_A_ORM_cpdag<-cpdag(dag_02_A_ORM,wlbl = TRUE)

graphviz.plot(dag_02_A_ORM_cpdag)

dag_02_B_ORM<-boot.strength(model_variables_02_ORM,R=200,algorithm = "hc",algorithm.args = list(blacklist=bl_02_byEthno_racial_group,whitelist=wl_02_byEthno_racial_group))

avg_dag_02_B_ORM<-averaged.network(dag_02_B_ORM)

strength.plot(avg_dag_02_B_ORM,dag_02_B_ORM)

plot(dag_02_B_ORM)

model_02_ORM_fitted<-bn.fit(avg_dag_02_B_ORM,model_variables_02_ORM)

**#Inference queries using learned Bayesian networks**

**##Querying proportion of each CMBCD stage for each combination of Age, Gender, Ethno_racial_group**

models <- list(

"non-Hispanic White"=model_02_NHW_fitted,

"non-Hispanic Black"=model_02_NHB_fitted,

"Hispanic"=model_02_HI_fitted,

"Other or Multiracial"=model_02_ORM_fitted)

CMBCD_query <- function(inputs, stage) {

evidence <- as.list(inputs)

model <- models[[evidence$Ethno_racial_group]]

evidence$Ethno_racial_group <- NULL

cpquery(model, evidence = evidence,

event = eval(bquote(CMBCD==.(stage))), method = "lw", n=10^7)

}

input_df <- expand.grid(

Age=levels(model_variables$Age),

Gender=levels(model_variables$Gender),

Ethno_racial_group=levels(model_variables$Ethno_racial_group)

)

CMBCD_df = data.frame(input_df)

for (stage in levels(model_variables$CMBCD)) {

cat("STAGE: ", stage, "\n")

results <- apply(input_df, 1, CMBCD_query, stage=stage)

CMBCD_df[[stage]] <- results

}

CMBCD_df

CMBCD_df<-melt(CMBCD_df,id.vars = c("Age","Gender","Ethno_racial_group"),measure.vars = c("Stage 0","Stage 1","Stage 2-4"))

CMBCD_df %>% ggplot(aes(x=Age,y=value,fill=variable))+

geom_bar(stat = "identity",position = "stack")+

facet_grid(Gender~Ethno_racial_group)+

ylab("Probability of CMBCD")+labs(fill="CMBCD Stage")+

scale_fill_brewer(palette = "Reds")+

geom_text_repel(aes(label=percent(value,accuracy = 1)),position="stack")

ggsave("../figures/CMBCD_byAgeGenderEthno_racial_group.png",width = 10,height = 5)

**##Querying proportion of each ABCD stage for each combination of Age, Gender, Ethno_racial_group**

models <- list(

"non-Hispanic White"=model_02_NHW_fitted,

"non-Hispanic Black"=model_02_NHB_fitted,

"Hispanic"=model_02_HI_fitted,

"Other or Multiracial"=model_02_ORM_fitted)

abcd_query <- function(inputs, stage) {

evidence <- as.list(inputs)

model <- models[[evidence$Ethno_racial_group]]

evidence$Ethno_racial_group <- NULL

cpquery(model, evidence = evidence,

event = eval(bquote(ABCD==.(stage))), method = "lw", n=10^7)

}

input_df <- expand.grid(

Age=levels(model_variables$Age),

Gender=levels(model_variables$Gender),

Ethno_racial_group=levels(model_variables$Ethno_racial_group)

)

abcd_df = data.frame(input_df) # so results don't feed back into the queries

for (stage in levels(model_variables$ABCD)) {

cat("STAGE: ", stage, "\n")

results <- apply(input_df, 1, abcd_query, stage=stage)

abcd_df[[stage]] <- results

}

abcd_df

abcd_df<-melt(abcd_df,id.vars = c("Age","Gender","Ethno_racial_group"),measure.vars = c("Stage 0","Stage 1","Stage 2","Stage 3","Stage 4"))

abcd_df %>% ggplot(aes(x=Age,y=value,fill=variable))+

geom_bar(stat = "identity",position = "stack")+

facet_grid(Gender~Ethno_racial_group)+

ylab("Probability of ABCD")+labs(fill="ABCD Stage")+

scale_fill_brewer(palette = "Reds")+

geom_text_repel(aes(label=percent(value,accuracy = 1)),position = "stack")

ggsave("../figures/ABCD_byAgeGenderEthno_racial_group.png",width = 10,height = 5)

**##Querying proportion of each DBCD stage for each combination of Age, Gender, Ethno_racial_group**

models <- list(

"non-Hispanic White"=model_02_NHW_fitted,

"non-Hispanic Black"=model_02_NHB_fitted,

"Hispanic"=model_02_HI_fitted,

"Other or Multiracial"=model_02_ORM_fitted)

DBCD_query <- function(inputs, stage) {

evidence <- as.list(inputs)

model <- models[[evidence$Ethno_racial_group]]

evidence$Ethno_racial_group <- NULL

cpquery(model, evidence = evidence,

event = eval(bquote(DBCD==.(stage))), method = "lw", n=10^7)

}

input_df <- expand.grid(

Age=levels(model_variables$Age),

Gender=levels(model_variables$Gender),

Ethno_racial_group=levels(model_variables$Ethno_racial_group)

)

DBCD_df = data.frame(input_df) # so results don't feed back into the queries

for (stage in levels(model_variables$DBCD)) {

cat("STAGE: ", stage, "\n")

results <- apply(input_df, 1, DBCD_query, stage=stage)

DBCD_df[[stage]] <- results

}

DBCD_df

DBCD_df<-melt(DBCD_df,id.vars = c("Age","Gender","Ethno_racial_group"),measure.vars = c("Stage 0","Stage 1","Stage 2","Stage 3","Stage 4"))

DBCD_df %>% ggplot(aes(x=Age,y=value,fill=variable))+

geom_bar(stat = "identity",position = "stack")+

facet_grid(Gender~Ethno_racial_group)+

ylab("Probability of DBCD")+labs(fill="DBCD Stage")+

scale_fill_brewer(palette = "Oranges")+

geom_text_repel(aes(label=percent(value,accuracy = 1)),position="stack")

ggsave("../figures/DBCD_byAgeGenderEthno_racial_group.png",width = 10,height = 5)

**##Querying proportion of each HBCD stage for each combination of Age, Gender, Ethno_racial_group**

models <- list(

"non-Hispanic White"=model_02_NHW_fitted,

"non-Hispanic Black"=model_02_NHB_fitted,

"Hispanic"=model_02_HI_fitted,

"Other or Multiracial"=model_02_ORM_fitted)

HBCD_query <- function(inputs, stage) {

evidence <- as.list(inputs)

model <- models[[evidence$Ethno_racial_group]]

evidence$Ethno_racial_group <- NULL

cpquery(model, evidence = evidence,

event = eval(bquote(HBCD==.(stage))), method = "lw", n=10^7)

}

input_df <- expand.grid(

Age=levels(model_variables$Age),

Gender=levels(model_variables$Gender),

Ethno_racial_group=levels(model_variables$Ethno_racial_group)

)

HBCD_df = data.frame(input_df) # so results don't feed back into the queries

for (stage in levels(model_variables$HBCD)) {

cat("STAGE: ", stage, "\n")

results <- apply(input_df, 1, HBCD_query, stage=stage)

HBCD_df[[stage]] <- results

}

HBCD_df

HBCD_df<-melt(HBCD_df,id.vars = c("Age","Gender","Ethno_racial_group"),measure.vars = c("Stage 0","Stage 1","Stage 2","Stage 3","Stage 4"))

HBCD_df %>% ggplot(aes(x=Age,y=value,fill=variable))+

geom_bar(stat = "identity",position = "stack")+

facet_grid(Gender~Ethno_racial_group)+

ylab("Probability of HBCD")+labs(fill="HBCD Stage")+

scale_fill_brewer(palette = "Blues")+

geom_text_repel(aes(label=percent(value,accuracy =1)),position="stack")

ggsave("../figures/HBCD_byAgeGenderEthno_racial_group.png",width = 10,height = 5)

**##Querying proportion of each LBCD stage for each combination of Age, Gender, Ethno_racial_group**

models <- list(

"non-Hispanic White"=model_02_NHW_fitted,

"non-Hispanic Black"=model_02_NHB_fitted,

"Hispanic"=model_02_HI_fitted,

"Other or Multiracial"=model_02_ORM_fitted)

LBCD_query <- function(inputs, stage) {

evidence <- as.list(inputs)

model <- models[[evidence$Ethno_racial_group]]

evidence$Ethno_racial_group <- NULL

cpquery(model, evidence = evidence,

event = eval(bquote(LBCD==.(stage))), method = "lw", n=10^7)

}

input_df <- expand.grid(

Age=levels(model_variables$Age),

Gender=levels(model_variables$Gender),

Ethno_racial_group=levels(model_variables$Ethno_racial_group)

)

LBCD_df = data.frame(input_df) # so results don't feed back into the queries

for (stage in levels(model_variables$LBCD)) {

cat("STAGE: ", stage, "\n")

results <- apply(input_df, 1, LBCD_query, stage=stage)

LBCD_df[[stage]] <- results

}

LBCD_df

LBCD_df<-melt(LBCD_df,id.vars = c("Age","Gender","Ethno_racial_group"),measure.vars = c("Stage 0","Stage 1","Stage 2","Stage 3","Stage 4"))

LBCD_df %>% ggplot(aes(x=Age,y=value,fill=variable))+

geom_bar(stat = "identity",position = "stack")+

facet_grid(Gender~Ethno_racial_group)+

ylab("Probability of LBCD")+labs(fill="LBCD Stage")+

scale_fill_brewer(palette = "Greens")+

geom_text_repel(aes(label=percent(value,accuracy = 1)),position="stack")

ggsave("../figures/LBCD_byAgeGenderEthno_racial_group.png",width = 10,height = 5)

**##Querying proportion of each ABCD stage for each combination of Age, Gender, Ethno_racial_group, Diet**

models <- list(

"non-Hispanic White"=model_02_NHW_fitted,

"non-Hispanic Black"=model_02_NHB_fitted,

"Hispanic"=model_02_HI_fitted,

"Other or Multiracial"=model_02_ORM_fitted)

abcd_query <- function(inputs, stage) {

evidence <- as.list(inputs)

model <- models[[evidence$Ethno_racial_group]]

evidence$Ethno_racial_group <- NULL

cpquery(model, evidence = evidence,

event = eval(bquote(ABCD==.(stage))), method = "lw", n=10^7)

}

input_df <- expand.grid(

Age=levels(model_variables$Age),

Gender=levels(model_variables$Gender),

Diet=levels(model_variables$Diet),

Ethno_racial_group=levels(model_variables$Ethno_racial_group)

)

abcd_diet_df = data.frame(input_df) # so results don't feed back into the queries

for (stage in levels(model_variables$ABCD)) {

cat("STAGE: ", stage, "\n")

results <- apply(input_df, 1, abcd_query, stage=stage)

abcd_diet_df[[stage]] <- results

}

**###Calculating relative change in probability of ABCD stage 4 with change in Diet for each combination of Age, Gender, Ethno_racial_group**

abcd_diet_df_copy<-abcd_diet_df

abcd_diet_df_RR<-abcd_diet_df_copy[,-c(5:8)]

abcd_diet_df_RR<-reshape(abcd_diet_df_RR,idvar = c("Age","Gender","Ethno_racial_group"),timevar = "Diet",direction = "wide")

abcd_diet_df_RR$Abs_diff<-abcd_diet_df_RR$`Stage 4.DASH-accordant`- abcd_diet_df_RR$`Stage 4.Not DASH-accordant`

abcd_diet_df_RR$Rel_diff<-abcd_diet_df_RR$Abs_diff/abcd_diet_df_RR$`Stage 4.Not DASH-accordant`

abcd_diet_df_RR$pos<-abcd_diet_df_RR$Rel_diff>=0

abcd_diet_df_RR %>%

ggplot(aes(x=Ethno_racial_group,y=Rel_diff))+geom_bar(stat = "identity",position = "identity",fill="red3")+ylab("Rel. Change in P(ABCD Stage 4) with Improved Diet")+

facet_grid(Gender~Age)+geom_hline(yintercept = 0)+

theme(axis.text.x = element_text(angle=30))+

geom_text(aes(label=percent(Rel_diff,accuracy = 1),vjust=ifelse(Rel_diff>=0,-0.1,1.1)))+

guides(fill="none")+theme(axis.title = element_text(size=14),axis.text = element_text(size=10),strip.text = element_text(size = 10))

ggsave("../figures/RR_Diet.png",width = 8,height = 6.5)

**##Querying proportion of each ABCD stage for each combination of Age, Gender, Ethno_racial_group, Physical activity (PA)**

models <- list(

"non-Hispanic White"=model_02_NHW_fitted,

"non-Hispanic Black"=model_02_NHB_fitted,

"Hispanic"=model_02_HI_fitted,

"Other or Multiracial"=model_02_ORM_fitted)

abcd_query <- function(inputs, stage) {

evidence <- as.list(inputs)

model <- models[[evidence$Ethno_racial_group]]

evidence$Ethno_racial_group <- NULL

cpquery(model, evidence = evidence,

event = eval(bquote(ABCD==.(stage))), method = "lw", n=10^7)

}

input_df <- expand.grid(

Age=levels(model_variables$Age),

Gender=levels(model_variables$Gender),

Physical_activity=levels(model_variables$Physical_activity),

Ethno_racial_group=levels(model_variables$Ethno_racial_group)

)

abcd_PA_df = data.frame(input_df) # so results don't feed back into the queries

for (stage in levels(model_variables$ABCD)) {

cat("STAGE: ", stage, "\n")

results <- apply(input_df, 1, abcd_query, stage=stage)

abcd_PA_df[[stage]] <- results

}

**###Calculating relative change in probability of ABCD stage 4 with change in Physical activity (PA) for each combination of Age, Gender, Ethno_racial_group**

abcd_PA_df_copy<-abcd_PA_df

abcd_PA_df_RR<-abcd_PA_df_copy[,-c(5:8)]

abcd_PA_df_RR<-reshape(abcd_PA_df_RR,idvar = c("Age","Gender","Ethno_racial_group"),timevar = "Physical_activity",direction = "wide")

abcd_PA_df_RR$Abs_diff<-abcd_PA_df_RR$`Stage 4.Sufficient`- abcd_PA_df_RR$`Stage 4.None`

abcd_PA_df_RR$Rel_diff<-abcd_PA_df_RR$Abs_diff/abcd_PA_df_RR$`Stage 4.None`

abcd_PA_df_RR$pos<-abcd_PA_df_RR$Rel_diff>=0

abcd_PA_df_RR %>%

ggplot(aes(x=Ethno_racial_group,y=Rel_diff))+geom_bar(stat = "identity",position = "identity",fill="red3")+ylab("Rel. Change in P(ABCD Stage 4) with Improved Physical Activity")+

facet_grid(Gender~Age)+geom_hline(yintercept = 0)+

theme(axis.text.x = element_text(angle=30))+ylim(-0.23,0.23)+

geom_text(aes(label=percent(Rel_diff,accuracy = 1),vjust=ifelse(Rel_diff>=0,-0.1,1.1)))+

guides(fill="none")+theme(axis.title = element_text(size=14),axis.text = element_text(size=10),strip.text = element_text(size = 10))

ggsave("../figures/RR_PA.png",width = 8,height = 6.5)

**##Querying proportion of each ABCD stage for each combination of Age, Gender, Ethno_racial_group, Smoking**

models <- list(

"non-Hispanic White"=model_02_NHW_fitted,

"non-Hispanic Black"=model_02_NHB_fitted,

"Hispanic"=model_02_HI_fitted,

"Other or Multiracial"=model_02_ORM_fitted)

abcd_query <- function(inputs, stage) {

evidence <- as.list(inputs)

model <- models[[evidence$Ethno_racial_group]]

evidence$Ethno_racial_group <- NULL

cpquery(model, evidence = evidence,

event = eval(bquote(ABCD==.(stage))), method = "lw", n=10^7)

}

input_df <- expand.grid(

Age=levels(model_variables$Age),

Gender=levels(model_variables$Gender),

Smoking_status=levels(model_variables$Smoking_status),

Ethno_racial_group=levels(model_variables$Ethno_racial_group)

)

abcd_smoking_df = data.frame(input_df) # so results don't feed back into the queries

for (stage in levels(model_variables$ABCD)) {

cat("STAGE: ", stage, "\n")

results <- apply(input_df, 1, abcd_query, stage=stage)

abcd_smoking_df[[stage]] <- results

}

**###Calculating relative change in probability of ABCD stage 4 with change in Smoking for each combination of Age, Gender, Ethno_racial_group**

abcd_smoking_df_copy<-abcd_smoking_df

abcd_smoking_df_RR<-abcd_smoking_df_copy[,-c(5:8)]

abcd_smoking_df_RR<-reshape(abcd_smoking_df_RR,idvar = c("Age","Gender","Ethno_racial_group"),timevar = "Smoking_status",direction = "wide")

abcd_smoking_df_RR$Abs_diff<-abcd_smoking_df_RR$`Stage 4.Never`- abcd_smoking_df_RR$`Stage 4.Current`

abcd_smoking_df_RR$Rel_diff<-abcd_smoking_df_RR$Abs_diff/abcd_smoking_df_RR$`Stage 4.Current`

abcd_smoking_df_RR$pos<-abcd_smoking_df_RR$Rel_diff>=0

abcd_smoking_df_RR %>%

ggplot(aes(x=Ethno_racial_group,y=Rel_diff))+geom_bar(stat = "identity",position = "identity",fill="red3")+ylab("Rel. Change in P(ABCD Stage 4) with Improved Smoking Status")+

facet_grid(Gender~Age)+geom_hline(yintercept = 0)+

theme(axis.text.x = element_text(angle=30))+ylim(-0.23,0.23)+

geom_text(aes(label=percent(Rel_diff,accuracy = 1),vjust=ifelse(Rel_diff>=0,-0.1,1.1)))+

guides(fill="none")+theme(axis.title = element_text(size=14),axis.text = element_text(size=10),strip.text = element_text(size = 10))

ggsave("../figures/RR_smoking.png",width = 8,height = 6.5)

**##Querying proportion of each ABCD stage for each combination of Age, Gender, Ethno_racial_group, Alcohol**

models <- list(

"non-Hispanic White"=model_02_NHW_fitted,

"non-Hispanic Black"=model_02_NHB_fitted,

"Hispanic"=model_02_HI_fitted,

"Other or Multiracial"=model_02_ORM_fitted)

abcd_query <- function(inputs, stage) {

evidence <- as.list(inputs)

model <- models[[evidence$Ethno_racial_group]]

evidence$Ethno_racial_group <- NULL

cpquery(model, evidence = evidence,

event = eval(bquote(ABCD==.(stage))), method = "lw", n=10^7)

}

input_df <- expand.grid(

Age=levels(model_variables$Age),

Gender=levels(model_variables$Gender),

Alcohol_use=levels(model_variables$Alcohol_use),

Ethno_racial_group=levels(model_variables$Ethno_racial_group)

)

abcd_alcohol_df = data.frame(input_df) # so results don't feed back into the queries

for (stage in levels(model_variables$ABCD)) {

cat("STAGE: ", stage, "\n")

results <- apply(input_df, 1, abcd_query, stage=stage)

abcd_alcohol_df[[stage]] <- results

}

**###Calculating relative change in probability of ABCD stage 4 with change in Alcohol for each combination of Age, Gender, Ethno_racial_group**

abcd_alcohol_df_copy<-abcd_alcohol_df

abcd_alcohol_df_RR<-abcd_alcohol_df_copy[,-c(5:8)]

abcd_alcohol_df_RR<-reshape(abcd_alcohol_df_RR,idvar = c("Age","Gender","Ethno_racial_group"),timevar = "Alcohol_use",direction = "wide")

abcd_alcohol_df_RR$Abs_diff<-abcd_alcohol_df_RR$`Stage 4.None`- abcd_alcohol_df_RR$`Stage 4.Heavy`

abcd_alcohol_df_RR$Rel_diff<-abcd_alcohol_df_RR$Abs_diff/abcd_alcohol_df_RR$`Stage 4.Heavy`

abcd_alcohol_df_RR$pos<-abcd_alcohol_df_RR$Rel_diff>=0

abcd_alcohol_df_RR %>%

ggplot(aes(x=Ethno_racial_group,y=Rel_diff))+geom_bar(stat = "identity",position = "identity",fill="red3")+ylab("Rel. Change in P(ABCD Stage 4) with Improved Alcohol Use")+

facet_grid(Gender~Age)+geom_hline(yintercept = 0)+

theme(axis.text.x = element_text(angle=30))+ylim(-0.23,0.23)+

geom_text(aes(label=percent(Rel_diff,accuracy = 1),vjust=ifelse(Rel_diff>=0,-0.1,1.1)))+

guides(fill="none")+theme(axis.title = element_text(size=14),axis.text = element_text(size=10),strip.text = element_text(size = 10))

ggsave("../figures/RR_alcohol.png",width = 8,height = 6.5)

References

1. Bickel G, Nord M, Price C, Hamilton W, Cook J. Guide to Measuring Household Food Security. 2000. <https://nhis.ipums.org/nhis/resources/FSGuide.pdf>.

2. Mellen PB, Gao SK, Vitolins MZ, Goff DC, Jr. Deteriorating dietary habits among adults with hypertension: DASH dietary accordance, NHANES 1988-1994 and 1999-2004. *Arch Intern Med* 2008; **168**(3): 308-14.

3. World Health Organization. Global Physical Activity Questionnaire (GPAQ) Analysis Guide: World Health Organization.

4. U.S. Department of Health and Human Services. Physical Activity Guidelines for Americans. Washington, DC; US: Department of Health and Human Services, 2018.

5. Gay IC, Tran DT, Paquette DW. Alcohol intake and periodontitis in adults aged >/=30 years: NHANES 2009-2012. *J Periodontol* 2018; **89**(6): 625-34.

6. Group SR, Wright JT, Jr., Williamson JD, et al. A Randomized Trial of Intensive versus Standard Blood-Pressure Control. *N Engl J Med* 2015; **373**(22): 2103-16.

7. Hall JE, do Carmo JM, da Silva AA, Wang Z, Hall ME. Obesity-induced hypertension: interaction of neurohumoral and renal mechanisms. *Circ Res* 2015; **116**(6): 991-1006.

8. Horita S, Seki G, Yamada H, Suzuki M, Koike K, Fujita T. Insulin resistance, obesity, hypertension, and renal sodium transport. *Int J Hypertens* 2011; **2011**: 391762.

9. Howard BV. Insulin resistance and lipid metabolism. *Am J Cardiol* 1999; **84**(1A): 28J-32J.

10. Klop B, Elte JW, Cabezas MC. Dyslipidemia in obesity: mechanisms and potential targets. *Nutrients* 2013; **5**(4): 1218-40.

11. Lean ME, Leslie WS, Barnes AC, et al. Primary care-led weight management for remission of type 2 diabetes (DiRECT): an open-label, cluster-randomised trial. *Lancet* 2018; **391**(10120): 541-51.

12. Miao Z, Alvarez M, Ko A, et al. The causal effect of obesity on prediabetes and insulin resistance reveals the important role of adipose tissue in insulin resistance. *PLoS Genet* 2020; **16**(9): e1009018.

13. Rossello X, Raposeiras-Roubin S, Oliva B, et al. Glycated Hemoglobin and Subclinical Atherosclerosis in People Without Diabetes. *J Am Coll Cardiol* 2021; **77**(22): 2777-91.

14. Tuomilehto J, Lindstrom J, Eriksson JG, et al. Prevention of type 2 diabetes mellitus by changes in lifestyle among subjects with impaired glucose tolerance. *N Engl J Med* 2001; **344**(18): 1343-50.
